# Pre-soil fumigation with ammonium bicarbonate and lime modulates the rhizosphere microbiome to mitigate clubroot disease in Chinese cabbage

**DOI:** 10.3389/fmicb.2024.1376579

**Published:** 2024-04-15

**Authors:** Jinhao Zhang, Xinghai Zhou, Yu Zhang, Zhenlin Dai, Zulei He, Yue Qiu, Sulaiman Ali Alharbi, Fangjun Wei, Lanfang Wei, Waqar Ahmed, Guanghai Ji

**Affiliations:** ^1^State Key Laboratory for Conservation and Utilization of Bio-Resources in Yunnan, Yunnan Agricultural University, Kunming, China; ^2^Key Laboratory of Agro-Biodiversity and Pest Management of Ministry of Education, Yunnan Agricultural University, Kunming, China; ^3^Plant Protection Research Institute, Guangxi Academy of Agricultural Science/Key Laboratory of Green Prevention and Control on Fruits and Vegetables in South China Ministry of Agriculture and Rural Affairs/Guangxi Key Laboratory of Biology for Crop Diseases and Insect Pests, Nanning Guangxi, China; ^4^Department of Botany and Microbiology College of Science, King Saud University, Riyadh, Saudi Arabia; ^5^Agricultural Foundation Experiment Teaching Center, Yunnan Agricultural University, Kunming, China; ^6^Guangdong Province Key Laboratory of Microbial Signals and Disease Control, College of Plant Protection, South China Agricultural University, Guangzhou, China

**Keywords:** *Plasmodiophora brassicae*, biological control, rhizosphere microbiome, disease suppression, soil pH

## Abstract

**Background:**

*Plasmodiophora brassicae* is an ever-increasing threat to cruciferous crop production worldwide.

**Aims and methods:**

This study investigated the impact of pre-soil fumigation with ammonium bicarbonate (N) and lime (NB) to manage clubroot disease in Chinese cabbage through 16S rRNA gene amplification sequencing.

**Results:**

We found that soil fumigation with N and NB suppressed disease incidence by reducing the soil acidity and population of *P. brassicae* in the rhizosphere. Minimum disease incidence and maximum relative control effect of about 74.68 and 66.28% were achieved in greenhouse and field experiments, respectively, under the combined application of ammonium bicarbonate and lime (LNB) as compared with N, NB, and control (GZ). Microbial diversity analysis through Miseq sequencing proved that pre-soil fumigation with N, NB, and LNB clearly manipulated rhizosphere microbial community composition and changed the diversity and structure of rhizosphere microbes compared with GZ. Bacterial phyla such as Proteobacteria, Bacteriodetes, and Acidobacteria and fungal phyla including Olpidiomycota and Ascomycota were most dominant in the rhizosphere of Chinese cabbage plants. Soil fumigation with N and NB significantly reduced the abundance of clubroot pathogen at genus (*Plasmodiophora*) level compared with GZ, while decreased further under combined application LNB. Microbial co-occurrence network analysis showed a highly connected and complex network and less competition for resources among microbes under combined application LNB.

**Conclusion:**

We conclude that for environmentally friendly and sustainable agriculture, soil fumigation with combined ammonium bicarbonate and lime plays a crucial role in mitigating Chinese cabbage clubroot disease by alleviating soil pH, reducing pathogen population, and manipulating the rhizosphere microbiome.

## 1 Introduction

*Plasmodiophora brassicae,* the causative agent of Clubroot disease, is a severe threat to cruciferous crop production globally as well as in China (Wei et al., 2021; Li et al., 2024). *P. brassicae* is broadly distributed in >60 countries and infects >300 plant species of the *Brassicaceae* family, resulting in 10–15% yield losses worldwide (Ren et al., 2016; He et al., 2023). In China, the average annual yield losses caused by *P. brassicae* are recorded at 20–30%. However, the areas of Yunnan, Chongqing, and Hubei are under a severe threat of yield reduction (Chai et al., 2014). Under high soil humidity and appropriate temperature, resting spores sprout, penetrate roots through root hairs and wounds, and multiply in the cortex so as to form root nodules, which hinder the plant nutrients and water absorbing capacity from the soil and results in stunted plant growth and death of the plant (Wei et al., 2021; Zhang et al., 2022a).

Due to its wide range of potential hosts and prolonged survival in the soil as a potentially fatal infection, the management of clubroot pathogens has become of utmost concern (Ahmed et al., 2020). Currently, the key approaches to control this devastating disease are crop rotation (Hwang et al., 2019; Yang et al., 2020), application of pesticides (Chai et al., 2014; Liu et al., 2019), and breeding of disease-resistant varieties (Song et al., 2016). The effects of pesticides are limited and have a negative impact on the environment and food safety concerns (Hu et al., 2021). The use of resistant varieties, because of the transformation of pathotypes of *Brassicaceae*, the resistance will be low, or the crop will lose its resistance owing to the highly infectious nature of the pathogen (Strelkov et al., 2018; Hollman et al., 2021). So, it is necessary to develop integrated disease management strategies to control this devastating disease.

Chemicals including calcium cyanamide, methyl bromide, methyl iodide, 1,3-dichloro propene, and propargyl bromide have been widely used as soil fumigants to manage soilborne diseases, insect pests, plant parasitic nematodes, and weeds for high-value production of crops (Luo et al., 2010). In China, due to environmental concerns, the usage of soil fumigants is banned (Wesemael et al., 2011) and was phased out in 2015 (Jiang et al., 2017). Thus, there is an urgent need to find a safe and reliable fumigation method in the form of cultural control to mitigate the incidence of soilborne diseases. Soil amendment with lime has been used as an important method to control clubroot incidence on cruciferous crops by increasing the soil pH and calcium contents and by improving the soil’s physicochemical properties (Murakami et al., 2002; Niwa et al., 2008). Similarly, soil fumigation with inorganic nitrogen is an alternate method to manage the occurrence of soilborne diseases (Wang et al., 2014).

Many previous studies reported that soil fumigation with lime and ammonium bicarbonate significantly alleviates soilborne diseases, especially fungal diseases, by decreasing the soil pH and restructuring the soil microbiome (Shen et al., 2018, 2019; Deng et al., 2021). Yet, little evidence is present on using ammonium bicarbonate and lime as soil fumigants to manage the clubroot disease of Chinese cabbage. So, keeping in mind the importance of soil fumigants, in this study, we investigated the impact of pre-soil fumigation with ammonium bicarbonate and lime on the occurrence of clubroot disease in Chinese cabbage and rhizosphere microbial diversity. We hypothesized that pre-soil fumigation with ammonium bicarbonate and lime mitigates the incidence of clubroot disease in Chinese cabbage by reducing the soil acidity and modulating the rhizosphere microbiome. We aimed to develop environmentally friendly and sustainable agricultural techniques for the efficient management of clubroot disease for the healthy production of cruciferous crops and to minimize the usage of fungicides.

## 2 Materials and methods

### 2.1 Site selection and plant material

The greenhouse and field experiments were executed in the greenhouse of “State Key Laboratory for Conservation and Utilization of Bio-resources in Yunnan,” Yunnan Agricultural University and Xiadabai County, Kunming (25° 2′ N and 102° 42′ E), China. The field is severely infected with clubroot pathogen, and Chinese cabbage has been grown in the field for >8 years. The annual rainfall and annual average temperature at Xiadabai County were recorded at 1,534 mm and 15.1°C, respectively. Chinese cabbage variety Luchunbai No. 1, highly susceptible to *P. brassicae,* was used as plant material.

### 2.2 Greenhouse experiment

The greenhouse experiment was performed from March to May 2021 under controlled conditions: 30/18°C day/night temperature and 10/14 h light/dark photoperiod (Zhang et al., 2022b). The pots (24 × 18 cm diameter) were filled with 3.5 kg/pot of diseased soil heavily infected with clubroot pathogen collected from the field (Xiadabai County, Kunming, China). Lime and ammonium bicarbonate were added to each pot at the ratio 1:1,300 (m/m), mixed thoroughly with the diseased soil, covered with plastic film (0.08 mm), and placed in a greenhouse at >35°C. The experiment was performed under four different conditions: NB; soil fumigation with lime (2.69 g/pot), N; soil fumigation with ammonium bicarbonate (2.69 g/pot), LNB; soil fumigation with combined ammonium bicarbonate (2.69 g/pot) and lime (2.69 g/pot), and GZ; no soil fumigation as control. After 15 days of anaerobic soil fumigation, Chinese cabbage seedlings were transplanted in pots (5 plants/pots). In addition, fertilizer was applied in each pot before the transplantation of seedlings to overcome the nutrient deficiency (Wei et al., 2021). The experiment was repeated three times with 15 plants per replication (in total, 45 plants/treatment) and was accomplished using a completely randomized design.

### 2.3 Assessment of control effect and soil pH

The control effect (CE), disease index (DI), and disease incidence (Di) were assessed at the end of the experiment, while soil pH was measured after soil fumigation and the end of the experiment. The Di, DI, and CE were calculated according to a five-point disease scoring scale and formulas (Zhang et al., 2022b). The soil pH was calculated in a 2.5:1 water/soil (V/W) suspension using a pH meter (Zhang et al., 2022a).

### 2.4 Field experiment

Field experiment was conducted during the growing season from June to August 2021 under four different situations: NB; soil fumigation with lime (0.15 kg/m^2^), N; soil fumigation with ammonium bicarbonate (0.15 kg/m^2^), LNB; soil fumigation with combined lime (0.15 kg/m^2^) and ammonium bicarbonate (0.15 kg/m^2^), and GZ; no soil fumigation as control. After soil amendments with ammonium bicarbonate and lime, the field was divided into plots (3 m × 9 m) and covered with 0.08 mm film for 15 days for anaerobic soil fumigation. The experiment was performed using a randomized complete block design and repeated three times with 3 plots/treatment (in total 12 plots), and each plot contained 120 plants of Chinese cabbage. The Di, DI, CE, and soil pH from each treatment were recorded at the completion of the trial as described above using the methodology of Zhang et al. (2022a).

### 2.5 Rhizosphere soil sampling and DNA extraction

Soil samples were collected in replicates (minimum three biological replicates/treatment) from each treatment from greenhouse and field experiments. Briefly, 5 plants per replication from each treatment were uprooted, and the excess soil was removed by shaking plants (Ahmed et al., 2022). Soil adjacent to the root surface was collected as rhizosphere soil samples for qPCR amplification and rhizosphere microbial diversity analysis. Briefly, five cores of soil samples from each replication were thoroughly mixed to make one composite sample, and three samples were collected from each treatment. Following the manufacturer’s instructions, 0.5 g of soil per sample was used to extract soil DNA using a PowerSoil^®^ DNA isolation Kit. In order to minimize the error, three cores of DNA were extracted from each sample, mixed thoroughly to make one sample, and kept at −80°C.

### 2.6 Analysis of *Plasmodiophora brassicae* population in the rhizosphere of Chinese cabbage plants

Quantitative PCR (qPCR) was amplified using the specific primer PB05−F (GAACCGGTCCACACACACACACACTAGAT) and PB05−R (GCCCACTGTGTTAATGATCC) to investigate *P. brassicae* population dynamics in the rhizosphere of Chinese cabbage plants. For qPCR amplification, each 20 μL reaction mixture contained 10 μL of 2× SuperReal PreMix Plus (SYBR Green I), 1.0 μL of PB05F/PB05R primers, 2 μL of DNA, and 7 μL of sterilized distilled water. The qPCR reaction was carried out in iCycler iQ5 (Bio-Rad, California, United States) thermal cycler under the following conditions: initial denaturation for 3 min at 95°C, with 40 cycles of denaturation for 10 s at 95°C, annealing for 30 s at 56°C, and extension for 20 s at 72°C.

### 2.7 Rhizosphere bacterial and fungal diversity analysis

PCR was performed for V3-V4 and ITS2 variable regions of 16S rRNA and ITS genes of bacteria and fungi with primer pairs 341F/806R and ITS5/ITS2, respectively, for analysis of rhizosphere microbial diversity (Ahmed et al., 2022, 2024). The obtained PCR products were purified using the AxyPrepDNAGel Extraction Kit (AXYGEN, United States) and sequenced on an Illumina MiSeq platform at Meggenomics Biotech Co., Ltd. (Guangdong, China) for the construction of paired-end reads.

### 2.8 Data processing

The paired-end reads collected from the Illumina MiSeq sequencer were initially processed through FLASH v1.2.11 to remove sequences (Magoč and Salzberg, 2011) and trimmed with Trimmomatic v0.36 at a score < 20 for quality control (Bolger et al., 2014). UCHIME v8.1 was used to remove the chimeras and for the production of high-quality reads (Edgar et al., 2011). Using the UPARSE pipeline, the reads were clustered into OTUs (operational taxonomic units) with a 97% similarity score (Edgar, 2013). The taxonomic annotation of fungal and bacterial OTUs was performed using the ribosomal database project (RDP) classifier v2.2 based on UNITE (Kõljalg et al., 2005) and SLIVA (Quast et al., 2012) databases, respectively.

### 2.9 Bioinformatics and statistical analyses

QIIME 2 was used to estimate the alpha and beta diversity metrics of bacterial and fungal communities (Bolyen et al., 2019). The beta diversity of bacterial and fungal communities was visualized using Principal Coordinates Analysis (PCoA) based on Weighted and Unweighted UniFrac distance metrics (Zhang et al., 2020). The differences in the composition of microbial communities were assessed by PERMANOVA using “Adonis” in R v4.2.0 (Anderson and Walsh, 2013). The relative abundance (RA) bar plots at phylum and genus levels and RA abundance heatmap at OTUs level for both bacterial and fungal communities were generated using R scripts in R v4.2.0. A co-occurrence network analysis was performed among the top 20 bacterial and fungal OTUs using the “sparcc package” and visualized by “psych package” in R (v.4.2.0) according to “Spearman correlation coefficient > 0.6 and *p* < 0.05.” Data were statistically examined using analysis of variance (ANOVA), and the least significant difference (LSD; *p* < 0.05) was used to assess the significant differences among treatments.

## 3 Results

### 3.1 Impact of soil fumigation on the occurrence of clubroot disease

**Figure 1:**
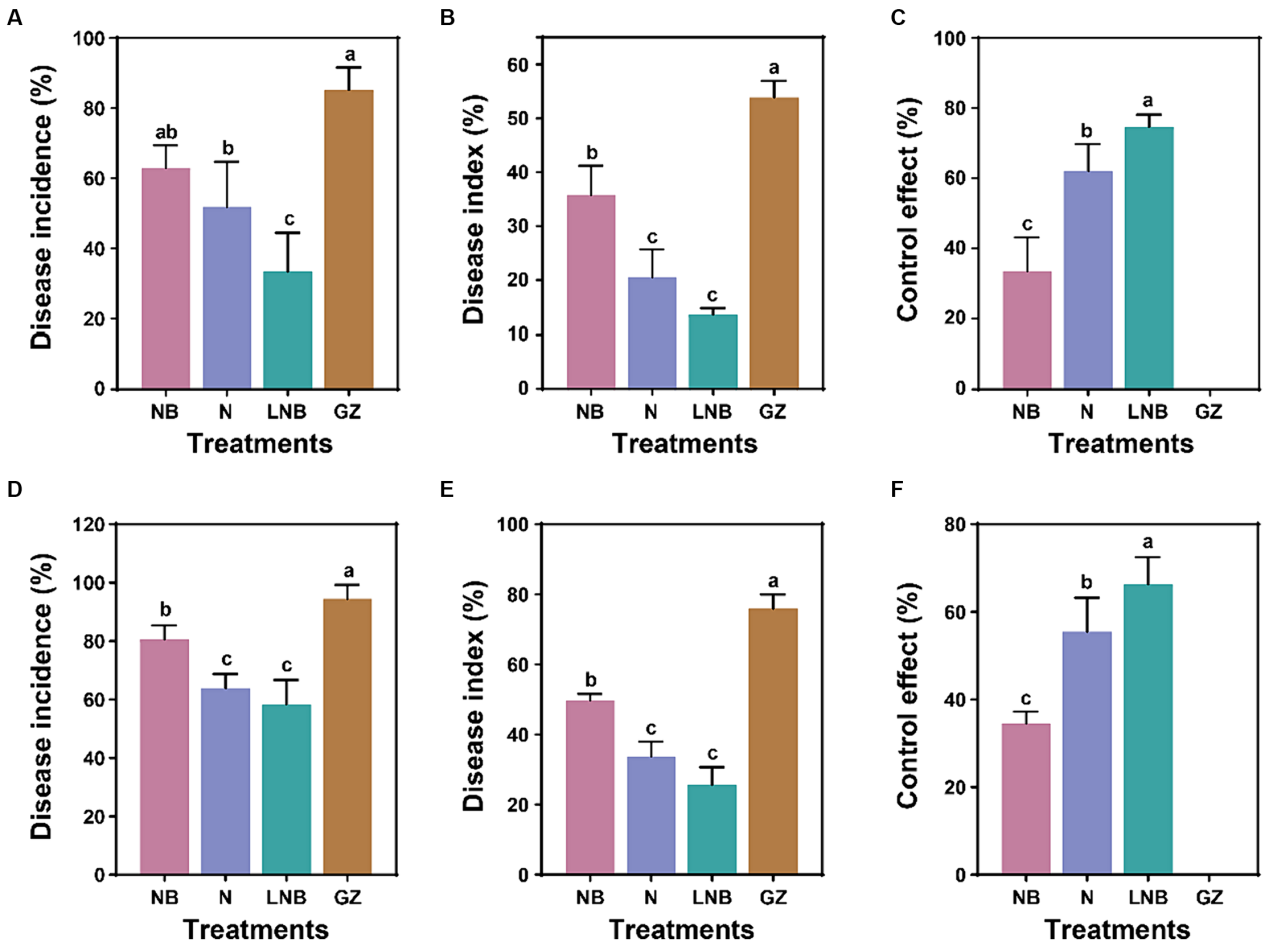
Impact of soil fumigation on clubroot disease of Chinese cabbage. Disease incidence **(A)**, disease index **(B)**, and control effect **(C)** under greenhouse conditions. Disease incidence **(D)**, disease index **(E)**, and control effect **(F)** under field conditions. Different small letters on the error bar show the significant difference among treatments according to the least significant difference (LSD) test at *p* < 0.05. Soil fumigation with lime (NB), soil fumigation with ammonium bicarbonate (N), combined soil fumigation with ammonium bicarbonate and lime (LNB), and non-fumigated soil (GZ).

Greenhouse and field experiments were performed to assess the effect of soil fumigation on the occurrence of Chinese cabbage clubroot disease (Figure 1; Supplementary Tables S1, S2). At the end of each experiment, the Di%, DI%, and CE% were calculated from each treatment using a disease rating scale and observation of gall formation on the roots. Results of greenhouse experiment demonstrated that soil fumigation with lime (NB) and ammonium bicarbonate (N) significantly reduced the incidence of clubroot of Chinese cabbage, having Di (62.96 and 51.85%), DI (35.80 and 20.57%), and CE (33.51 and 62.10%), respectively compared to control (GZ) Di (85.19%), DI (53.88%), and CE (0%) (Figures 1A–C). However, minimum Di (33.33%) and DI (13.58%), and highest CE (74.68%) were achieved under the combined application of ammonium bicarbonate and lime (LNB) compared with NB, N, and GZ (Figures 1A–C).

A field experiment was conducted to further examine the potential of soil fumigation to mitigate clubroot incidence in Chinese cabbage under natural conditions (Figures 1D–F). Results of field experiment revealed that soil fumigation with NB and N significantly alleviate clubroot incidence in Chinese cabbage having Di (80.55 and 63.89%), DI (49.69 and 33.64%), and CE (34.48 and 55.45%), respectively than control (GZ) Di (94.45%), DI (75.93%), and CE (0%) (Figures 1D–F). Whereas, combined application of ammonium bicarbonate and lime (LNB) results in minimum values of Di (58.33%) and DI (25.62%) compared with NB, N, and GZ, and the relative control effect of LNB on clubroot of cabbage can be reached up to 66.28% (Figures 1D–F). These results suggested that the application of ammonium bicarbonate (N) and lime (NB) significantly reduced the Chinese cabbage clubroot disease under both greenhouse and field conditions. However, maximum results were obtained under the combined application of ammonium bicarbonate and lime (LNB).

### 3.2 Effect of soil fumigation on soil pH

**Figure 2:**
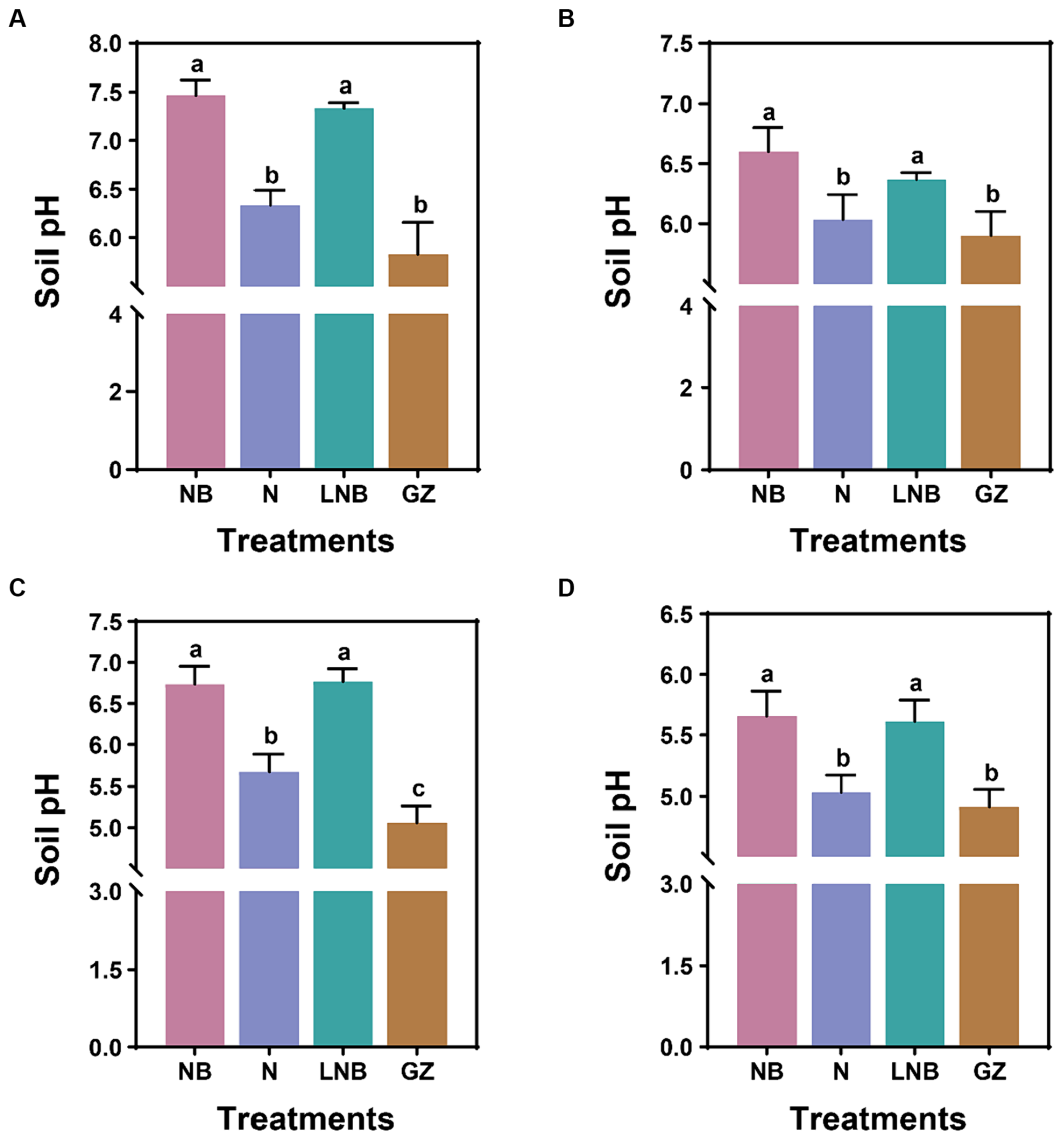
Effect of soil fumigation on soil pH. Soil pH after 15 days of fumigation **(A)** and after harvesting the crop **(B)** in the greenhouse experiment. Soil pH after 15 days of fumigation **(C)** and after harvesting the crop **(D)** in the field experiment. Significance difference among treatments (as described in Figure 1) is shown by different small letters on the error bar according to the least significant difference (LSD) test at *p* < 0.05.

We explored the effect of N, NB, and LNB as soil fumigants on soil pH after fumigation (after 15 days) and at the end of experiments (Figure 2; Supplementary Tables S1, S2). In the greenhouse experiment, after 15 days of soil fumigation with NB, N, LNB, and GZ, the soil pH values were recorded as 7.4, 6.3, 7.3, and 5.8, respectively. The soil pH was significantly increased under the application of NB and LNB and was found to be significantly higher than N and GZ, whereas there is no significance among NB and LNB (Figure 2A). At the end of experiment, after harvesting the Chinese cabbage crop, the values of soil pH under different treatments NB, N, LNB, and GZ were recorded as 6.6, 6.0, 6.4, and 5.9, respectively (Figure 2B). In the field experiment, 15 days after soil fumigation with NB, N, LNB, and GZ, the soil pH values were recorded as 6.7, 5.7, 6.8, and 5.1, respectively (Figure 2C). The values of soil pH were significantly increased under the application of NB, N, and LNB compared to GZ, while there was no significant difference between NB and LNB (Figure 2C). At the end of experiment, the values of soil pH under different treatments NB, N, LNB, and GZ were recorded as 5.7, 5.0, 5.6, and 4.9, respectively (Figure 2D). The results demonstrated that soil fumigation with NB and LNB significantly decreased the soil acidity compared with N and GZ.

### 3.3 Population dynamics of *Plasmodiophora brassicae*

**Figure 3:**
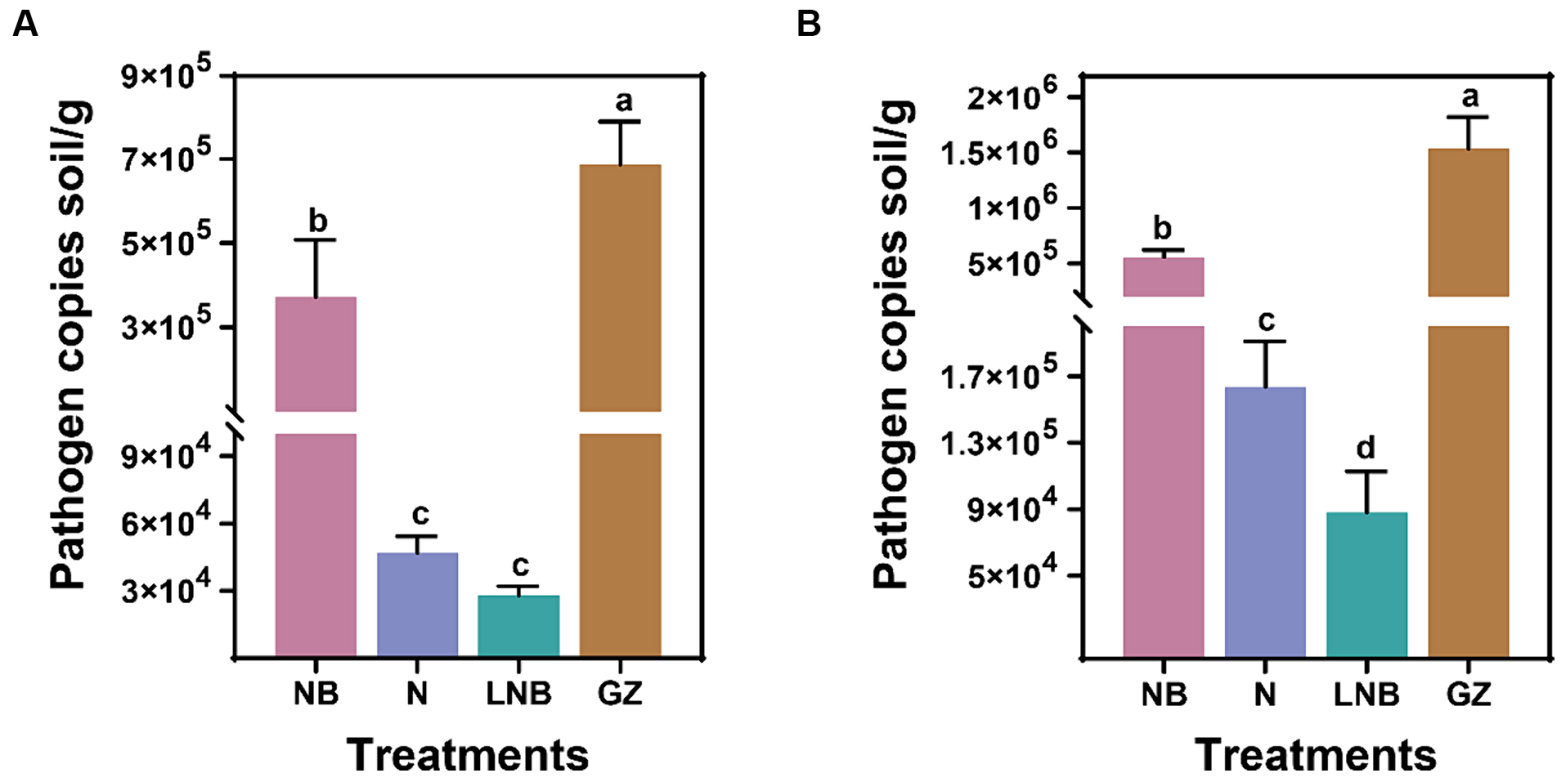
Population dynamic of clubroot pathogen *Plasmodiophora brassicae* in rhizosphere soil of Chinese cabbage plants under different treatments. *P. brassicae* copies per gram of soil under greenhouse **(A)** and field **(B)** conditions. According to the least significance test (LSD; *p* < 0.05), the different lowercase letters on the error bars showed a significant difference among treatments (as described in Figure 1).

We determined the effect of soil fumigation with N, NB, LNB, and GZ on the population dynamics of *P. brassicae* in the rhizosphere of Chinese cabbage in the greenhouse and field experiments. The presence of *P. brassicae* was confirmed through the amplification of standard qPCR, and population dynamics (copies per gram of soil) were calculated using a standard curve. Results of qPCR analysis showed that soil fumigation with N, NB, and LNB significantly reduced the gene copy number of *P. brassicae* per gram of soil compared with control (GZ) in the greenhouse and field experiments (Figure 3). The density of *P. brassicae* decreased from 7 × 10^5^ to 3 × 10^4^ copies per gram of soil and 1.5 × 10^6^ to 9 × 10^4^ copies per gram of soil under greenhouse and field experiments, respectively (Figures 3A,B). In the greenhouse experiment, the density of *P. brassicae* decreased in the order CK (7 × 10^5^) > NB (4 × 10^5^) > N (5 × 10^4^) ≥ LNB (3 × 10^4^) (Figure 3A). While under field conditions, the density of *P. brassicae* decreased in the order CK (1.5 × 10^6^) > NB (5 × 10^5^) > N (1.6 × 10^5^) > LNB (9 × 10^4^) (Figure 3B). These results suggest that the combined application of ammonium bicarbonate and lime (LNB) significantly decreased the density of *P. brassicae* in the rhizosphere of Chinese cabbage compared to that of NB, N, and CK.

### 3.4 Soil fumigation affects the microbiome assembly of Chinese cabbage

The microbiome assembly of Chinese cabbage changed significantly under four treatments (NB, N, LNB, and GZ). In total, 12 rhizosphere soil samples were subjected through the Illumina MiSeq platform for amplification of V3-V4 and ITS2 regions of bacteria and fungi, respectively, which resulted in a total of 1,513,933 (ave; 126,161/sample) and 1,064,123 (ave; 88,677/sample) raw reads, respectively (Supplementary Table S3). After quality control and chimera’s removal 1,396,930 (ave; 116,411/sample) and 1,003,248 (ave; 83,604/sample) high-quality reads were obtained for bacteria and fungi, respectively which were then clustered into 46,743 (ave; 3,895/sample) bacterial operational taxonomic units (OTUs) and fungal 5,627 (ave; 469/sample) OTUs for taxonomic annotation at 3% cutoff level (Supplementary Table S3).

### 3.5 Effect of soil fumigation on the structure and diversity of the microbial community

**Figure 4:**
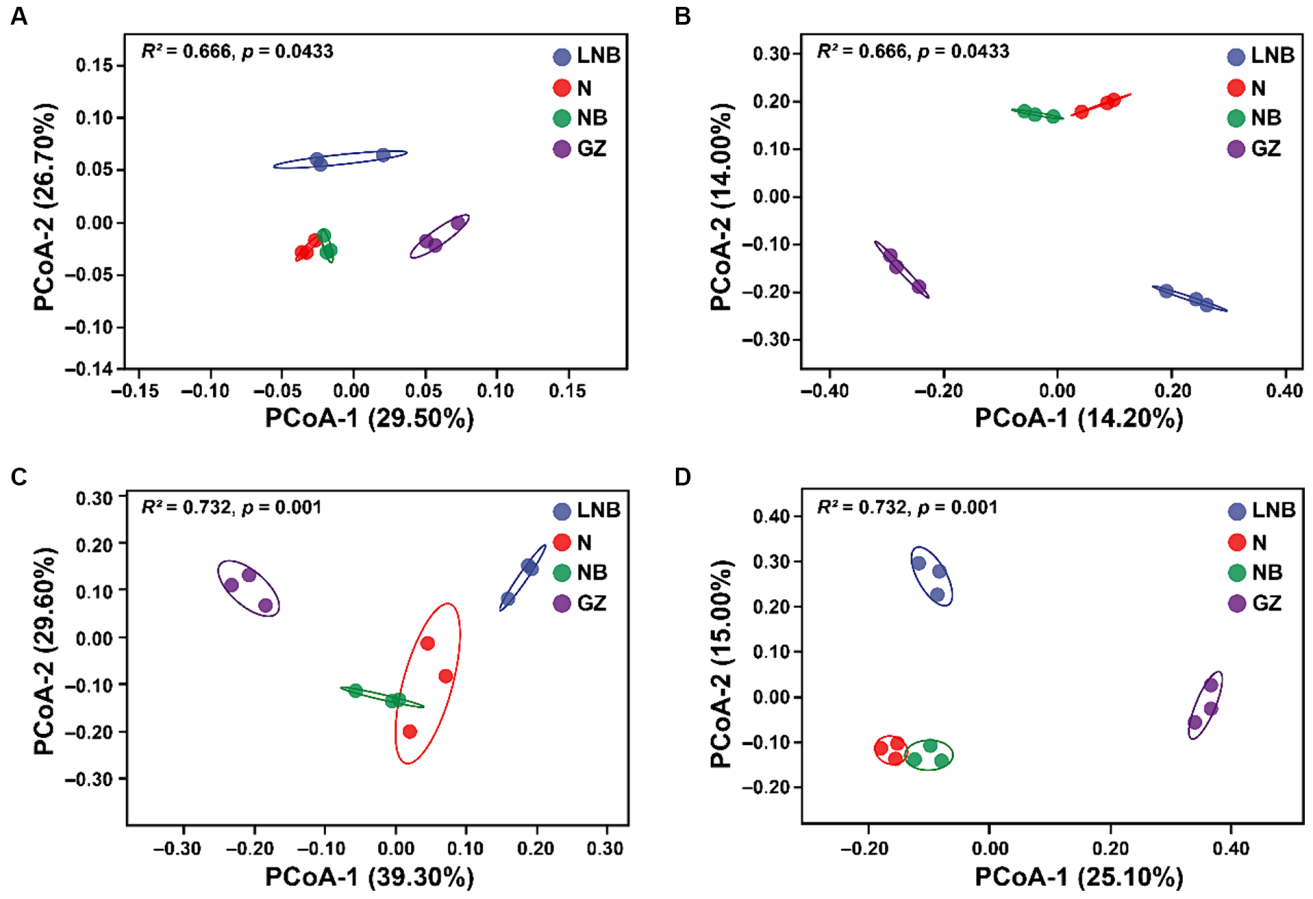
Principal coordinate analysis (PCoA) based on the Bray–Curtis dissimilarity metric showing the beta diversity metrics of rhizosphere bacterial and fungal communities under different treatments. Weighted and Unweighted UniFrac PCoA analysis of rhizosphere bacterial **(A,B)** and fungal **(C,D)** community structure under different treatments (as described in Figure 1).

Principal coordinate analysis (PCoA) based on Weighted UniFrac (abundance of taxa) and Unweighted UniFrac (sensitive to rare taxa) distance metrics was used for the visualization of changes in the structure (beta diversity) of microbial communities (Figure 4). According to PCoA results, the first 2-axis of Weighted UniFrace and Unweighted UniFrace showed a total of 56.20 and 28.20% variation for bacterial communities, respectively (Figures 4A,B) and 68.90 and 40.01% variation for fungal communities, respectively (Figures 4C,D). Further, PERMANOVA of pairwise interaction between treatments for bacterial (*R*^2^ = 0.666, *p* = 0.0433) and fungal (*R*^2^ = 0.732, *p* = 0.001) communities demonstrated that structure of rhizosphere microbiome significantly changed under different treatments (Supplementary Table S4). However, we cannot observe a significant difference in alpha diversity indices (Chao-1, Shannon, and Simpson) of bacterial and fungal communities under different treatments (Supplementary Table S5).

**Figure 5:**
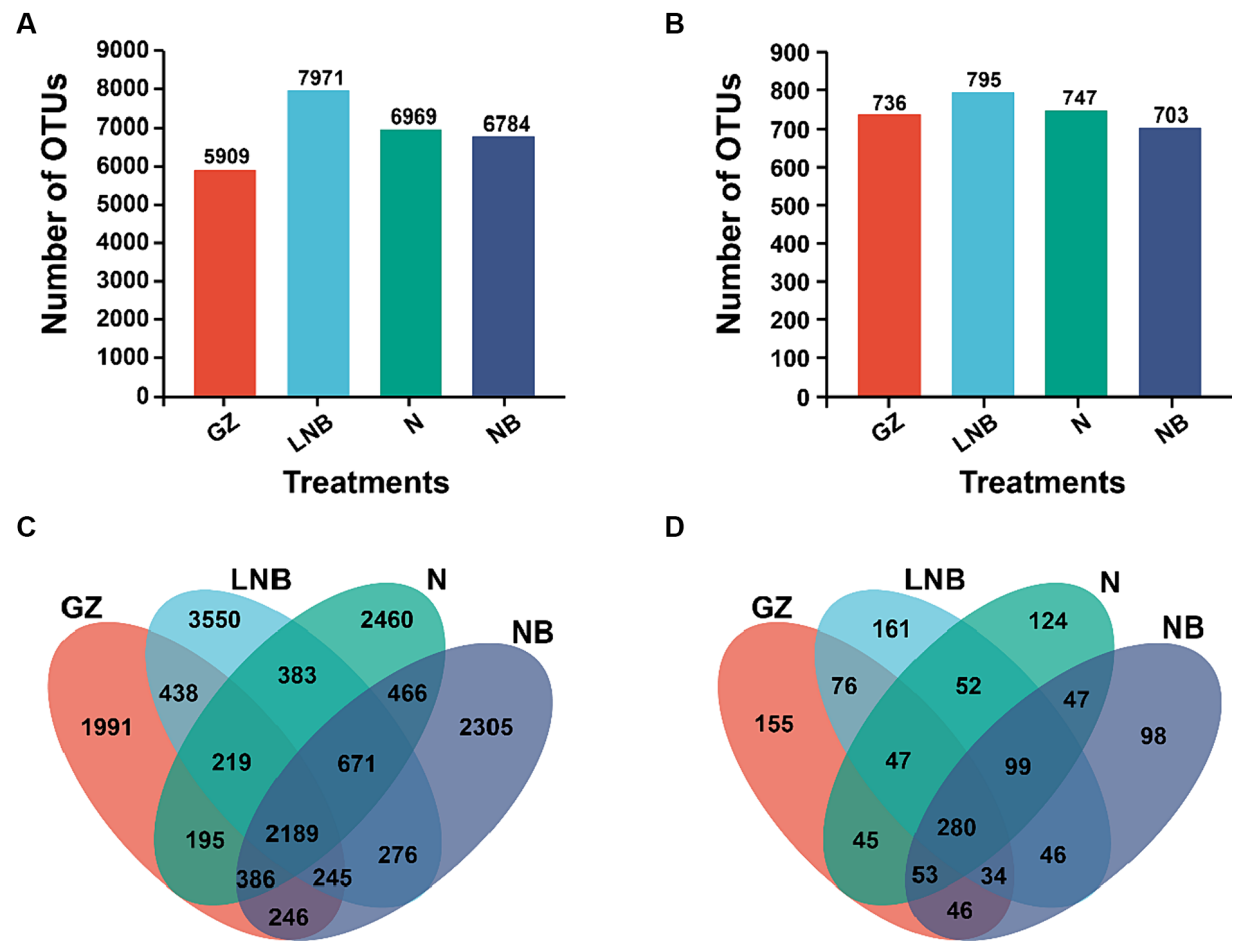
Operational taxonomic units (OTUs) analysis of bacterial and fungal communities under different treatments. Bar plots displaying the total numbers of bacterial **(A)** and fungal **(B)** OTUs under different treatments (as described in Figure 1). Venn diagram showing the unique and shared bacterial **(C)** and fungal **(D)** OTUs among the treatments (as described in Figure 1).

In addition, an OTUs analysis was performed for the taxonomic annotation of bacterial and fungal recovered OTUs under different treatments (Figure 5). OTUs analysis of different treatments demonstrated that a total of 5,909, 7,971, 6,969, and 6,784 bacterial and 736, 795, 747, and 703 fungal annotated OTUs were obtained from treatments GZ, LNB, N, and NB, respectively (Figures 5A,B). A Venn diagram further showed a variation among the bacterial and fungal annotated OTUs. According to the results of the Venn diagram, a total of 2,189 and 280 common bacterial and fungal OTUs, respectively, and 1991, 3,550, 2,460, and 2,305 bacterial and 155, 161, 124, and 98 fungal unique OTUs were recovered under different treatments GZ, LNB, N, and NB, respectively (Figures 5C,D). These results suggested that the changes in the microbial community structure might be due to the variation in the common and unique OTUs among the treatments.

### 3.6 Soil fumigation influences the microbial community composition at the phylum level

**Figure 6:**
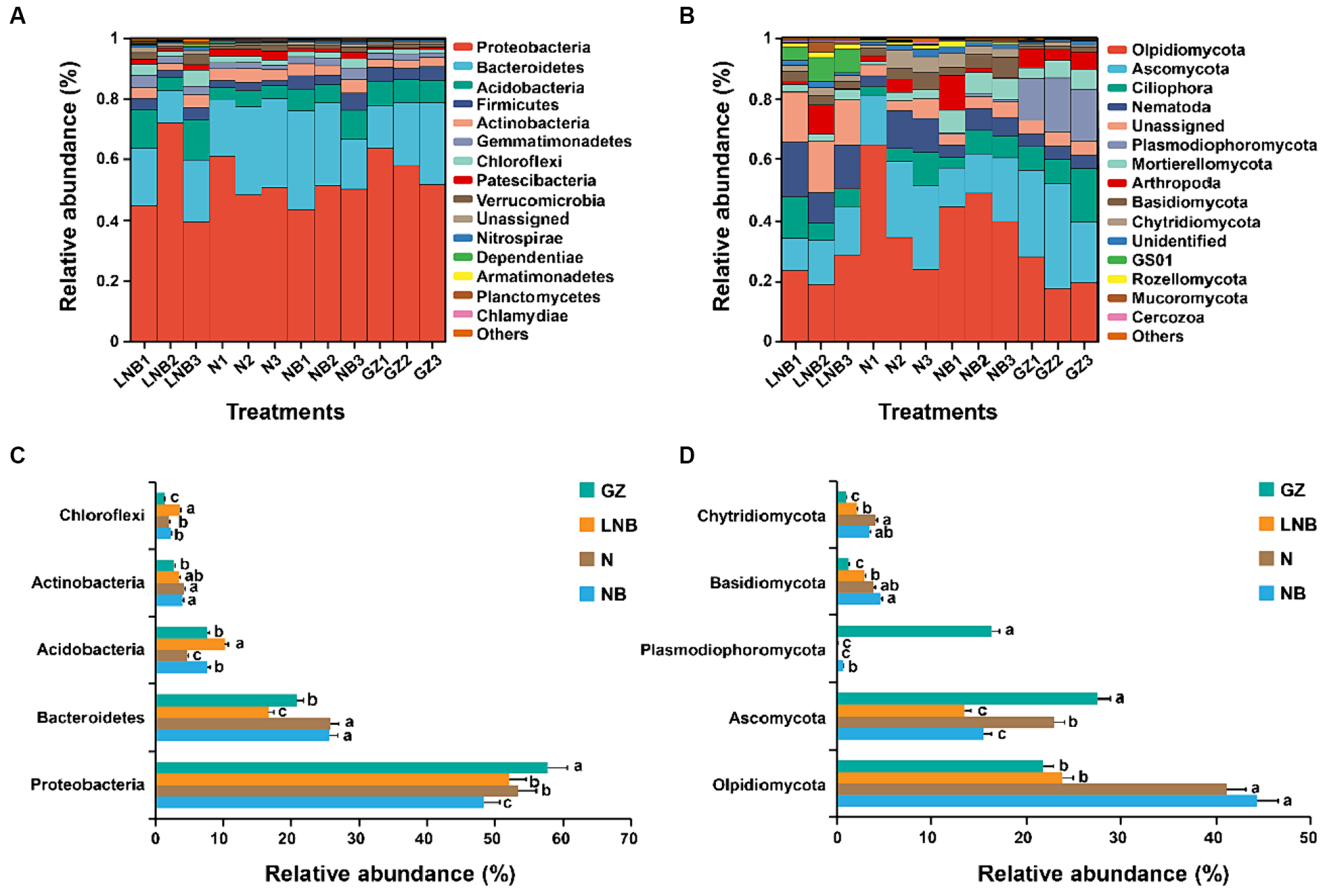
Relative abundance (RA) analysis of the most abundant bacterial and fungal phyla in the rhizosphere soil of Chinese cabbage. RA of top 15 bacterial **(A)** and fungal **(B)** phylum under different treatments. The bar plot shows the significant difference in RA of the differentially abundant bacterial **(C)** and fungal **(D)** phyla under different treatments. The significant difference in the RA of specific phylum among treatments (as described in Figure 1) is shown by different lowercase letters on the error bars according to the least significance difference test (LSD) *p* < 0.05.

Relative abundance (RA) analysis at the phylum level showed that bacterial and fungal community composition significantly changed under different treatments. The RA of the top 15 bacterial and fungal phyla in the rhizosphere of Chinese cabbage under different treatments is shown in Figure 6 and Supplementary Tables S6, S7. In all rhizosphere soil samples, Acidobacteria, Actinobacteria, Bacteroidetes, Firmicutes, and Proteobacteria were the dominant bacterial phyla, accounting for ~89.59% of total RA (Figure 6A; Supplementary Table S6). While Ascomycota, Basidiomycota, Chytridiomycota, Mortierellomycota, Olpidiomycota, and Plasmodiophoromycota were the dominant fungal phyla accounting for ~67.02 of total RA (Figure 6B; Supplementary Table S7). Bacterial phylum Proteobacteria was present in high RA with GZ, and its RA decreased in an order GZ > N ≥ LNB > NB (Figure 6C). The RA of Bacteroidetes was significantly higher in N and NB than LNB and GB (*p* < 0.05), while Acidobacteria and Chloroflexi were present in high RA in LNB compared with N, NB, and GZ (Figure 6C). Fungal phylum, including Olpidiomycota, Basidiomycota, and Chytridiomycota, were present in higher RA in N and NB than in LNB and GZ (Figure 6D). However, Ascomycota and Plasmodiophoromycota were the dominant fungal phyla in the rhizosphere of GZ compared to N, NB, and LNB. The RA of Ascomycota was decreased in an order GZ > N > NB ≥ LNB. The clubroot pathogen *P. brassicae* belongs to phylum Plasmodiophoromycota, which was present in 0.69, 0.06, 0.08, and 16.30% average RA in NB, N, LNB, and GZ (Supplementary Table S7), and its RA decreased as GZ > NB > LNB ≥ N (Figure 6D). The results showed that soil fumigation with N, NB, and LNB significantly decreased the RA of Plasmodiophoromycota as compared with non-fumigated soil (GZ).

### 3.7 Impact of soil fumigation on microbial community composition at the genus level

**Figure 7:**
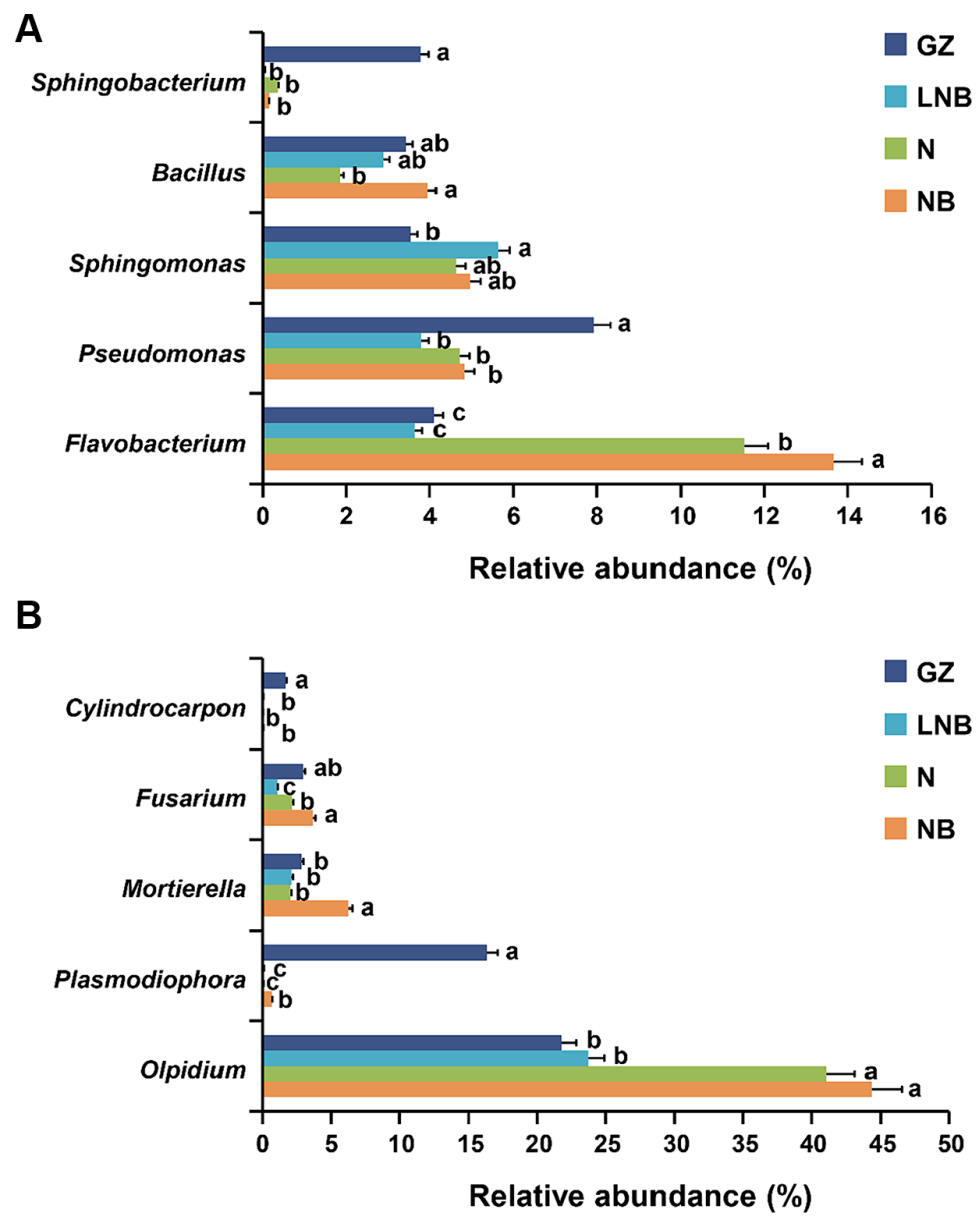
Relative abundance (RA) bar plots of most abundant genera under different treatments. RA bar plots of bacterial **(A)** and fungal **(B)** genera level. Significant difference in the RA of specific genera among the treatments (as described in Figure 1) is shown by different small letters on the error bar according to the least significant difference (LSD) test at *p* < 0.05.

The patterns of taxonomic distribution of bacterial and fungal communities at the genus level become more evident. Results demonstrated that bacterial and fungal community composition significantly changed at the genus level under different treatments. The RA abundance of the top 15 bacterial and fungal genera is shown in Supplementary Tables S8, S9. *Flavobacterium* was the most dominant bacterial genus in the rhizosphere of Chinese cabbage under different treatments, having an average RA of ~8.23%, and its RA decreased in an order NB > N > GZ ≥ LNB (Figure 7A; Supplementary Table S8). The RA of *Sphingobacterium* and *Pseudomonas* significantly increased in the rhizosphere of GZ compared to that of N, NB, and LNB. In contrast, *Sphingomonas* was found in higher RA in N, NB, and LNB than in GZ, and *Bacillus* was found in high RA with GZ, NB, and LNB compared with N (Figure 7A). *Olpidium* was the most dominant fungal genera with an average RA of about ~32.74% in the rhizosphere of Chinese cabbage under different treatments, and its RA decreased in an order NB ≥ N > LNB ≥ GZ (Figure 7B; Supplementary Table S9). *Fusarium* was dominant in NB and GZ more than N and LNB, while *Mortierella* was dominant in NB more than N, LNB, and GZ (Figure 7B). The RA of plant pathogenic genera such as *Cylindrocarpon* and *Plasmodiophora* significantly decreased after soil fumigation with N, NB, and LNB as compared to non-fumigation soil (GZ). The RA of genus *Cylindrocarpon* lowered in an order GZ > N, NB, and LNB, while the RA of *Plasmodiophora* was reduced in an order GZ > NB > N and LNB (Figure 7B). These results suggested that soil fumigation with lime and ammonium bicarbonate significantly reduced the population dynamics of the genus *Plasmodiophora,* the causative agent of clubroot of Chinese cabbage, and also reduced the RA of other harmful fungal genera pathogenic to plants. The results of microbial diversity analysis at the genus level are similar to the results of qPCR gene copy number, which showed that the population of *P. brassicae* significantly decreased under soil fumigation (N, NB, and LNB) than non-soil fumigation (GZ).

### 3.8 Assessment of specific bacterial and fungal OTUs in the rhizosphere of Chinese cabbage

**Figure 8:**
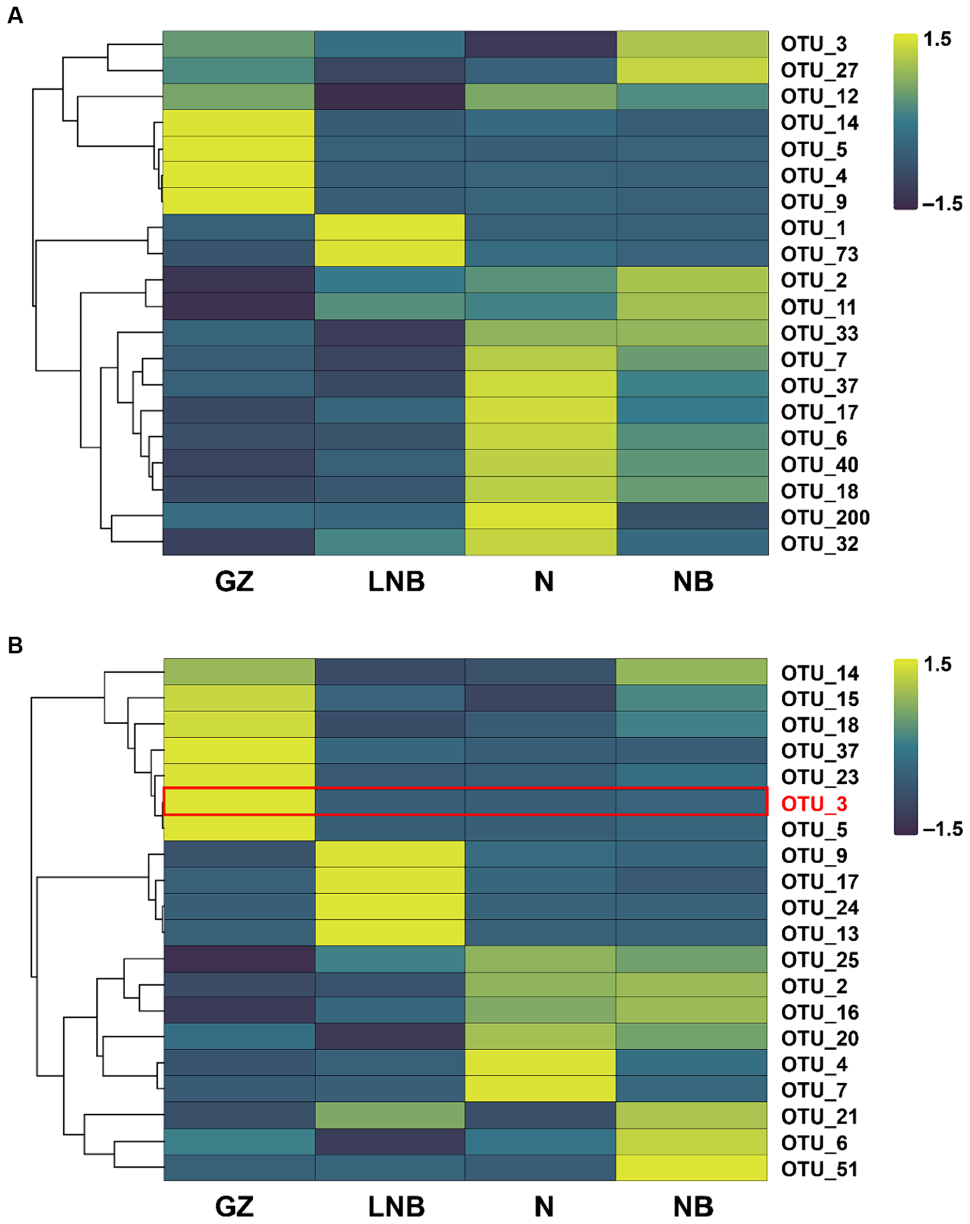
Relative abundance heatmaps for the top 20 bacterial **(A)** and fungal **(B)** OTUs in the rhizosphere of Chinese cabbage plants under different treatments (as described in Figure 1).

An OTU enrichment analysis was carried out to determine which bacterial and fungal taxa are sensitive to specific treatments. A total of 20 bacterial and 20 fungal-specific OTUs were recovered (RA > 0.01%) in the rhizosphere of Chinese cabbage under different treatments (Figure 8; Supplementary Tables S10, S11). A number of bacterial OTUs including OTU_7, OTU_37, OTU_17, OTU_6, OTU_40, OTU_18, OTU_200, and 32 were highly abundant to N followed by OTU_14, OTU_5, OTU_4, and OTU_9 dominant to GZ, OTU_3, OTU_27, OTU_2, and OTU_11 enriched to GZ and OTU_1 and OTU_73 dominant to LNB (Figure 8A; Supplementary Table S10). In the fungal community, OTUs such as OTU_14, OTU_15, OTU_18, OTU_37, OTU_23, OTU_3, and OTU_5 was predominant to GZ, while OTU_9, OTU_17, OTU_24, and OTU_13 was present in high RA with LNB. OTU_21, OTU_6, and OTU_51 were highly abundant in the rhizosphere soil of NB, and OTU_4 and OTU_7 were most dominant to LNB (Figure 8B; Supplementary Table S11).

The highly abundant bacterial OTUs were identified as *Providencia, Sphingomonas, Pseudomonas, Bacillus, Pedobacter, Flavobacterium, Acidovorax, Stenotrophomonas, Sphingobacterium, Bradyrhizobium, Pseudarthrobacter, Devosia,* and *Allorhizobium-Neorhizobium-Pararhizobium-Rhizobium*. The most abundant fungal OTUs have belonged to *Olpidium*, *Plasmodiophora*, *Sordariomycetes*, *Plectosphaerellaceae*, *Fusarium*, and *Mortierella*. It is worth noticing that OTU_3 was present in high RA in GZ and was identified as *P. brassicae*, while its RA significantly decreased in N, NB, and LNB (Figure 8B; Supplementary Table S11). These results demonstrated that soil fumigation with lime and ammonium bicarbonate significantly reduced the population of *P. brassicae* in the rhizosphere of Chinese cabbage, which mitigate the incidence of clubroot disease.

### 3.9 Soil fumigation influences the microbial co-occurrence network properties

**Figure 9:**
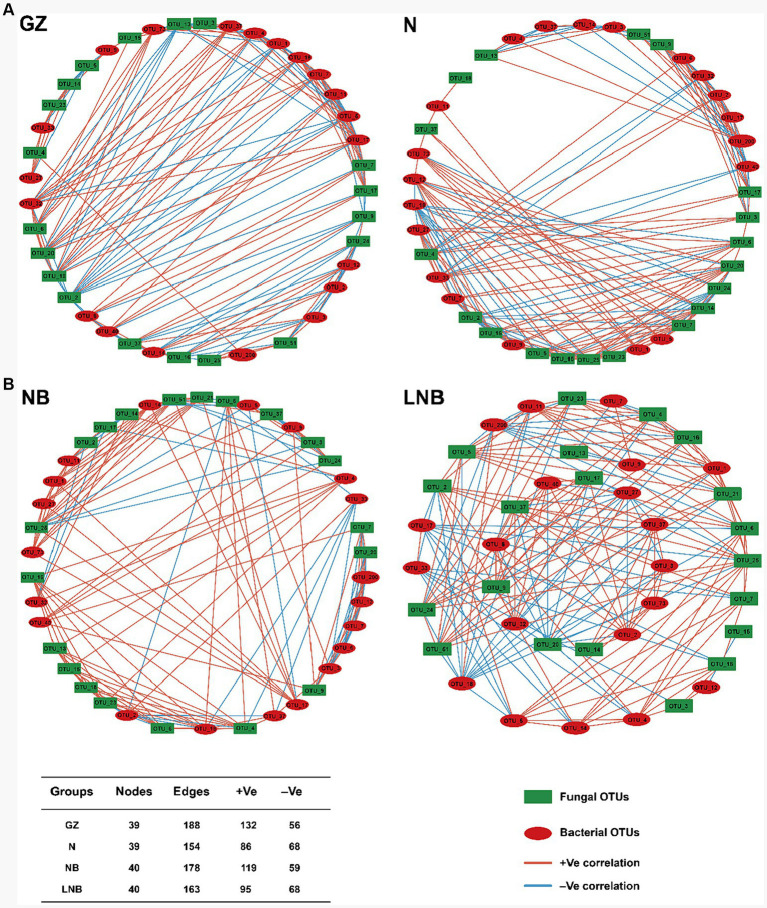
Co-occurrence network of bacterial and fungal communities under different treatments (as described in Figure 1) at the OTU level. Only *p* < 0.05 and corrections coefficients >0.6 (Spearman correlation) were constructed in the network. Red circles represent the bacterial OTUs, and green boxes represent the fungal OTUs. Red and blue edges indicate positive and negative correlations, respectively. Collinear network analysis of relationships between samples and species. Collinear network analysis of relationships between samples with bacteria phyla **(A)**. Collinear network analysis of relationships between samples with fungal phyla **(B)**.

A microbial co-occurrence network analysis was conducted among the top 20 bacterial and fungal OTUs according to “Spearman correlation coefficient” to investigate the overall correlations among the microbial communities (Figure 9). In network analysis, nodes represent the core bacterial and fungal OTUs, while edges show the positive and negative correlation among the microbial OTUs. The highest positive correlation was observed among the clubroot pathogen and rhizosphere microbes in GZ (70.21%) than that of NB (66.85%), N (55.84%), and LNB (58.28%). This suggested that rhizosphere microbes enhance the population of *P. brassicae* and competition for resources among microbes, which results in the highest disease incidence. In contrast, soil fumigation with N and LNB reduced the population of *P. brassicae* and competition for resources, which mitigates the incidence of clubroot by making the rhizosphere microbiome healthier and more complex.

## 4 Discussion

Clubroot caused by *P. brassicae* is one of the most destructive soilborne diseases affecting Chinese cabbage production all over China (Wei et al., 2021). Recently, clubroot disease has become more severe due to the increased continuous cropping and acidic nature of soils, which has led to a significant decrease in the yield of cruciferous crops (He et al., 2019). Currently, the prevention and control measures of clubroot disease mainly rely on the application of pesticides. However, the long-term and excessive use of pesticides leads to environmental pollution and provokes food safety concerns. Thus, there is an urgent need to find a new, effective, and stable control measure for the management and prevention of clubroot disease.

Previous studies have reported that soil fumigation with lime and ammonium bicarbonate alleviates the incidence of soilborne diseases such as *Fusarium* wilt of cucurbits (Sun et al., 2015), *Fusarium* wilt of banana (Shen et al., 2018), Panama disease of banana (Shen et al., 2019), and bacterial wilt of tomato (Deng et al., 2021). However, there is no report on the use of lime and ammonium bicarbonate as soil fumigants to alleviate the incidence of clubroot in Chinese cabbage. In this study, for the first time, we found that soil fumigation with lime (NB) and ammonium bicarbonate (N) significantly suppressed the incidence of clubroot disease in Chinese cabbage compared with non-fumigated soil (CK). However, a maximum control effect was achieved when soil fumigation was done with combined application of lime and ammonium bicarbonate (LNB) and our results are similar with the findings of previous reports as mentioned above.

It has been reported that soil acidification has exacerbated the outbreak of clubroot (Bhattacharya et al., 2014) and bacterial wilt (Li et al., 2022), and alleviating soil acidity significantly mitigates the incidence of soil-borne diseases (Zhang et al., 2022; Liu et al., 2023). Soil pH is one of the most significant abiotic environmental factors, correlates with the occurrence of soilborne disease, and is inextricably linked to plant health (Zhang et al., 2022a; Liu et al., 2023). Previous studies demonstrated that the incidence of clubroot is positively correlated with soil pH, and under acidic soil, the incidence of clubroot increased significantly (Zhang et al., 2022a). We found that soil fumigation with NB, N, and LNB significantly suppressed the incidence of clubroot disease by increasing the soil pH 6.77, 5.68, and 6.77, respectively, compared with CK (pH = 5.06). We found that the effect can be maintained throughout the growth period of Chinese cabbage. These results are consistent with previous reports that soil amendment with lime and ammonium bicarbonate increased the soil pH (Li et al., 2016; Shen et al., 2018). Some of the common integrated disease management approaches, such as soil amendments with biochar, rock dust, and lime, have been adopted to control soilborne disease by increasing the soil pH > 6.0 (Li and Dong, 2013; Zhang et al., 2017; Shen et al., 2019).

In addition, we found that soil fumigation with N, NB, and LNB significantly reduced the population dynamics of *P. brassicae* in the rhizosphere of Chinese cabbage compared with CK, which directly alleviated the incidence of clubroot disease. These results are similar to the previous reports that soil amendment with lime reduced the density of *P. brassicae* resting spores and disease severity (Murakami et al., 2002; Niwa et al., 2008; Shen et al., 2019). This might be due to the fact that lime can increase soil pH and calcium ion concentration and reduce the infection ability of *P. brassicae* to complete its primary life cycle, which affects the germination of resting spores, production of primary zoospores, and root hair infection (Murakami et al., 2002). Additionally, an alkaline pH encourages the synthesis of ammonium into ammonia, which can damage membrane integrity and reverse proton gradients across cell membranes (Shen et al., 2019). However, we cannot find a significant difference in soil pH between soil fumigation with NB and LNB. Thus, these results confirmed that lime plays a leading role in decreasing soil acidity, which further strengthens the ammonium bicarbonate potential to mitigate *P. brassicae* infection.

The ecological balance of soil microorganisms is the premise to ensure healthy plant growth. Soil fumigation with ammonium bicarbonate and lime can directly influence pathogens, improve the soil environment by decreasing soil acidity, and reshape soil microbial community structure, thereby directly or indirectly preventing and controlling soilborne diseases (Liu et al., 2016; Deng et al., 2021). It has been reported that soil fumigation altered soil microflora in plant monoculture systems by changing soil microbial community structure and stimulating the potentially beneficial microbial consortia (Li et al., 2016; Wang et al., 2023, 2024). The structure of microbial communities separates clearly under fumigated (N, NB, and LNB) and non-fumigated (GZ) treatments as revealed by PCoA based on Weighted Unifrac and Unweighted Unifrac analysis. The changes in the structure of rhizosphere microbial communities in fumigated and non-fumigated soil might be due to the changes in the shared and unique OTUs, as shown by the Venn diagrams, and each treatment recruit’s unique microbial communities. This suggested that the habitat of microbial communities significantly changed after soil fumigation with ammonium bicarbonate and lime, which is similar to previous reports that the application of lime and ammonium bicarbonate regulates the composition of rhizosphere microbial communities (Shen et al., 2018).

Ascomycota, Basidiomycota, Acidobacteria, Actinobacteria, Bacteroidetes, Firmicutes, and Proteobacteria are the most abundant fungal and bacterial phyla in the rhizosphere of field corps and agricultural soils (Delgado-Baquerizo et al., 2018; Ahmed et al., 2022; Liu et al., 2024). In this study, we found that bacterial phyla such as Proteobacteria, Bacteroidetes, Acidobacteria, Actinobacteria, and Firmicutes and fungal phyla including Ascomycota, Basidiomycota, and Olpidiomycota were dominant in the rhizosphere of Chinese cabbage across all samples. The application of lime and ammonium bicarbonate significantly increased the relative abundance of Actinobacteria and Chloroflexi compared with the control group (GZ). The members of Actinobacteria and Chloroflexi are well-known to have properties of disease suppression (Trivedi et al., 2017) and degradation of complex organic compounds to low molecular weight substances (Speirs et al., 2019). In this study, we found that the diversity of fungal communities was significantly reduced compared with bacterial communities in the fumigated soil; this might be due to the depletion of ammonium-sensitive fungal species. Our study confirms the results of previous findings that soil fumigation with chloroform, lime, and ammonium bicarbonate reduced the abundance of fungal communities (Griffiths et al., 2000; Shen et al., 2018). Interestingly, we observed that the abundance of clubroot pathogen *P. brassicae* significantly reduced at OTU, phylum, and genus levels in the fumigated soil as compared with non-fumigated soil. This was further proved by the analysis of qPCR and population (per gram of soil) of *P. brassicae* decreased in an order GZ (1.5 × 10^6^) > NB (5 × 10^5^) > N (1.6 × 10^5^) > LNB (9 × 10^4^).

Furthermore, the results of microbial co-occurrence network analysis revealed that the complexity of the co-occurrence network is closely related to soil pH. This study showed that soil fumigation with lime and ammonium bicarbonate decreased the soil acidity, which suppressed the population dynamics of *P. brassicae* in the rhizosphere of Chinese cabbage. Further, provides a favorable environment for the growth of beneficial microorganisms, which makes the network more complex by reducing the competition for resources. This is in line with previous studies demonstrated that microbial interaction is of great significance in restricting pathogen propagation, and in a highly complex network, microbial communities combat well with the pathogen through niche exclusion and limit the pathogen invasion (Huang et al., 2019; Liu et al., 2023).

## 5 Conclusion

**Figure 10:**
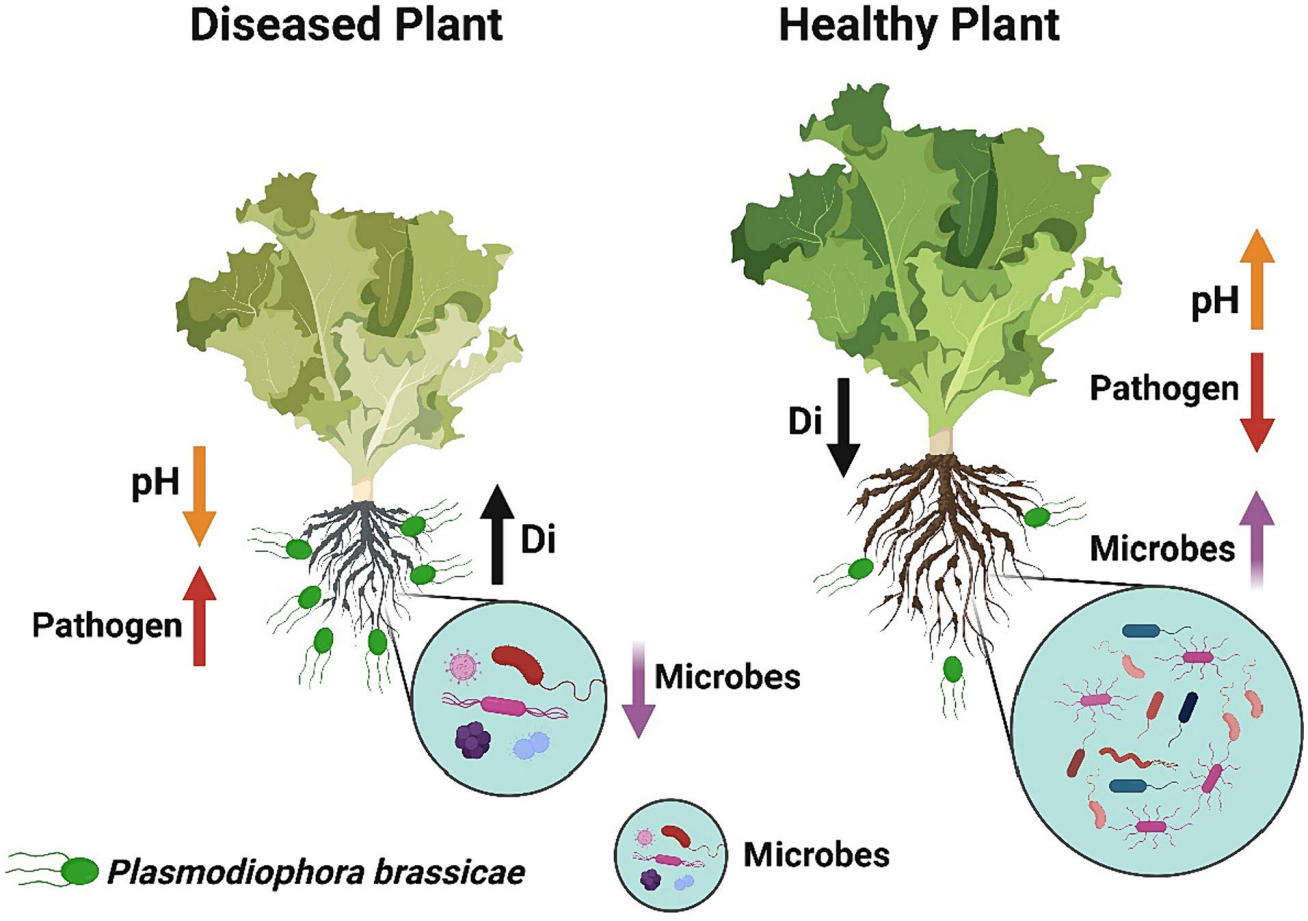
The concluding sketch demonstrates the impact of soil fumigation on the occurrence of clubroot disease and the rhizosphere microbiome of Chinese cabbage plants. Diseased plants under no soil fumigation display high disease incidence, high pathogen population, low microbes, and low soil pH. Healthy plants under lime and ammonium bicarbonate soil fumigation represent high soil pH, high microbes, low pathogen population, and low disease incidence.

In summary, we conclude that soil fumigation with ammonium bicarbonate and lime may serve as a substitute way to manage Chinese cabbage clubroot disease in green agriculture. However, the maximum control effect was achieved under the combined application of ammonium bicarbonate and lime. The suppression in disease incidence might be achieved because soil fumigation with ammonium bicarbonate and lime improves the soil pH. More precisely, we observed that soil fumigation with ammonium bicarbonate and lime (Figure 10): (1) Decreases the soil acidity, which is suitable for the growth of beneficial microorganisms and also provides an unfavorable environment for the development of clubroot pathogen *P. brassicae*, (2) Reduces the population dynamics of *P. brassicae* in the rhizosphere of Chinese cabbage plants, (3) Modifies the diversity, structure, and composition of rhizosphere microbial communities, and (4) reduces the competition for resources among microbes, which makes the rhizosphere microbiome healthier and more complex. This work enhanced our understanding of the primary role of ammonium bicarbonate and lime soil fumigation in mitigating *P. brassicae* infection in Chinese cabbage. Future studies will focus on identifying the impact of ammonium bicarbonate and lime soil fumigation on soil biochemical properties and functional parameters to anticipate disease suppression.

## Data availability statement

The datasets presented in this study can be found in online repositories. The datasets generated for this study can be found in the NCBI public database. All sequences of ITS and 16S rRNA genes can be found in Sequence Read Archive (SRA) under BioProject No. PRJNA967811.

## Ethics statement

All authors have been personally and actively involved in substantial work leading to the paper and will take public responsibility for its content.

## Author contributions

JZ: Conceptualization, Data curation, Formal analysis, Investigation, Methodology, Writing – original draft. XZ: Conceptualization, Data curation, Formal analysis, Writing – original draft. YZ: Formal analysis, Software, Validation, Writing – original draft. ZD: Conceptualization, Data curation, Methodology, Software, Writing – original draft. ZH: Data curation, Methodology, Validation, Writing – original draft. YQ: Data curation, Formal analysis, Software, Writing – original draft. FW: Data curation, Investigation, Validation, Writing – original draft. LW: Project administration, Supervision, Writing – original draft, Writing – review & editing. WA: Data curation, Formal analysis, Project administration, Software, Supervision, Validation, Writing – original draft, Writing – review & editing. GJ: Conceptualization, Funding acquisition, Project administration, Resources, Supervision, Writing – original draft, Writing – review & editing. SA: Formal analysis, Writing-review & editing.
