# Supplementary material for: Pre-soil fumigation with ammonium bicarbonate and lime modulates the rhizosphere microbiome to mitigate clubroot disease in Chinese cabbage
Source: Front Microbiol. 2024 Apr 15;15:1376579. doi: 10.3389/fmicb.2024.1376579 (PMC11057235; doi:10.3389/fmicb.2024.1376579)
Supplement: Supplementary file 1 [file Data_Sheet_1.docx]

**Pre-soil fumigation with ammonium bicarbonate and lime modulates the rhizosphere microbiome to mitigate clubroot disease in Chinese cabbage**

Jinhao Zhang^1,2#^, Xinghai Zhou^1,2#^, Yu Zhang^1,2^, Zhenlin Dai^1,2^, Zulei He^1,2^, Yue Qiu^1,2^, Sulaiman Ali Alharbi^5^, Fangjun Wei^1,2^, Lanfang Wei^1,3^*, Waqar Ahmed^4^*, and Guanghai Ji^1,2^*

^1^State Key Laboratory for Conservation and Utilization of Bio-resources in Yunnan, Yunnan Agricultural University, Kunming 650201, Yunnan, China

^2^Key Laboratory of Agro-Biodiversity and Pest Management of Ministry of Education, Yunnan Agricultural University, Kunming 650201, Yunnan, China

^3^Agricultural Foundation Experiment Teaching Center, Yunnan Agricultural University, Kunming 650201, Yunnan, China

^4^Guangdong Province Key Laboratory of Microbial Signals and Disease Control, College of Plant Protection, South China Agricultural University, Guangzhou 510642, Guangdong, China.

^5^Department of Botany & Microbiology College of Science, King Saud University, Riyadh 11451, Saudi Arabia

^#^These authors contributed equally to this work.

***Correspondence:**

Lanfang Wei

wlfang2000@aliyun.com

Waqar Ahmed

ahmed.waqar1083@yahoo.com

Guanghai Ji

jghai001@163.com

| **Table S1:** Effect of soil fumigation on the disease incidence, disease index, and control effect on clubroot and soil pH of Chinese cabbage plants in the greenhouse. | | | | | |
| --- | --- | --- | --- | --- | --- |
| Treatment | Disease incidence (%) | Disease index (%) | Control effect (%) | Soil pH after fumigation | Soil pH after cabbage harvest |
| NB | 62.96±6.42ab | 35.80±5.38b | 33.51±9.62c | 7.47±0.15a | 6.60±0.20a |
| N | 51.85±12.83b | 20.57±5.15c | 62.10±7.61b | 6.33±0.15b | 6.03±0.21b |
| LNB | 33.33±11.11c | 13.58±1.25c | 74.68±3.45a | 7.33±0.06a | 6.37±0.06a |
| GZ | 85.19±6.41a | 53.88±3.06a |  | 5.83±0.32b | 5.90±0.20b |
| **Here,** soil fumigation with lime **(NB)**, soil fumigation with ammonium bicarbonate **(N)**, combined soil fumigation with ammonium bicarbonate and lime **(LNB)**, and non-fumigated soil **(GZ)**. | | | | | |

| **Table S2:** Effect of soil fumigation on the disease incidence, disease index, and control effect on clubroot and soil pH of Chinese cabbage plants in the field. | | | | | |
| --- | --- | --- | --- | --- | --- |
| Treatment | Disease incidence (%) | Disease index (%) | Control effect (%) | Soil pH after fumigation | Soil pH after cabbage harvest |
| NB | 80.55±4.81b | 49.69±1.93b | 34.48±2.77c | 6.73±0.22a | 5.66±0.21a |
| N | 63.89±4.82c | 33.64±4.38c | 55.45±7.76b | 5.68±0.20b | 5.03±0.14b |
| LNB | 58.33±8.34c | 25.62±5.10c | 66.28±6.22a | 6.77±0.16a | 5.61±0.18a |
| GZ | 94.45±4.81a | 75.93±4.03a |  | 5.06±0.21c | 4.91±0.15b |
| **Here,** soil fumigation with lime **(NB)**, soil fumigation with ammonium bicarbonate **(N)**, combined soil fumigation with ammonium bicarbonate and lime **(LNB)**, and non-fumigated soil **(GZ)**. | | | | | |

| **Table S3:** Sequencing data processing for variable regions of bacteria (16S; V3-V4) and fungi (ITS2) rRNA. | | | | | | | |
| --- | --- | --- | --- | --- | --- | --- | --- |
| **Sample ID** | **Raw reads** | **Clean reads** | **Clean tags** | **Q20 (%)** | **Q30 (%)** | **GC (%)** | **OTUs** |
| **Bacteria (16S; V3-V4)** | | | | | | | |
| NB1 | 129521 | 128508 | 119427 | 98.5 | 94.9 | 53.6 | 3782 |
| NB2 | 122326 | 121247 | 112986 | 98.5 | 94.7 | 53.8 | 3663 |
| NB3 | 121003 | 120193 | 112580 | 98.6 | 95.2 | 54.6 | 4188 |
| N1 | 127938 | 126734 | 118830 | 98.6 | 95 | 53.9 | 3970 |
| N2 | 127378 | 126365 | 117170 | 98.4 | 94.6 | 53.6 | 3968 |
| N3 | 129276 | 128398 | 119927 | 98.6 | 95.1 | 53.4 | 3894 |
| LNB1 | 120795 | 119990 | 111120 | 98.5 | 95 | 54.8 | 4665 |
| LNB2 | 128115 | 127141 | 117802 | 98.5 | 94.7 | 54.4 | 3757 |
| LNB3 | 131608 | 130617 | 121179 | 98.6 | 95 | 54.7 | 4871 |
| GZ1 | 122172 | 121273 | 112934 | 98.6 | 95 | 54.4 | 3342 |
| GZ2 | 125931 | 124921 | 115411 | 98.4 | 94.6 | 53.7 | 3364 |
| GZ3 | 127870 | 126803 | 117564 | 98.4 | 94.6 | 53.6 | 3279 |
| Total | 1,513,933 | 1,502,190 | 1,396,930 |  |  |  | 46,743 |
| Average | 126,161 | 125,183 | 116,411 |  |  |  | 3,895 |
| **Fungi (ITS1)** | | | | | | | |
| NB1 | 86558 | 86151 | 81341 | 99.2 | 96.7 | 50.5 | 412 |
| NB2 | 91233 | 90755 | 86718 | 99.3 | 97 | 49.1 | 449 |
| NB3 | 87367 | 87004 | 82487 | 99.3 | 97.2 | 50.1 | 456 |
| N1 | 85375 | 84878 | 80298 | 99.1 | 96.5 | 47.7 | 445 |
| N2 | 92397 | 91905 | 85482 | 99.2 | 96.8 | 50.3 | 458 |
| N3 | 87802 | 87420 | 82209 | 99.4 | 97.3 | 50.6 | 514 |
| LNB1 | 91245 | 90833 | 86422 | 99.4 | 97.4 | 51.2 | 514 |
| LNB2 | 85904 | 85565 | 81780 | 99.5 | 97.5 | 51.7 | 486 |
| LNB3 | 91585 | 91126 | 86368 | 99.3 | 97.1 | 51.4 | 465 |
| GZ1 | 85480 | 85068 | 80616 | 99.4 | 97.3 | 50.3 | 471 |
| GZ2 | 89444 | 89059 | 84596 | 99.5 | 97.6 | 51.6 | 455 |
| GZ3 | 89733 | 89311 | 84931 | 99.4 | 97.5 | 51 | 502 |
| Total | 1,064,123 | 1,059,075 | 1,003,248 |  |  |  | 5,627 |
| Average | 88,677 | 88,256 | 83,604 |  |  |  | 469 |
| **Here,** soil fumigation with lime **(NB)**, soil fumigation with ammonium bicarbonate **(N)**, combined soil fumigation with ammonium bicarbonate and lime **(LNB)**, and non-fumigated soil **(GZ)**. | | | | | | | |

| **Table S4:** Results of permutational multivariate analysis of variance (PERMANOVA) for bacterial and fungal communities. | | | | | | |
| --- | --- | --- | --- | --- | --- | --- |
| **Paris** | ***Df*** | **Sums of Sqs** | **Mean Sqs** | **F. Model** | ***R^2^*** | ***p*** |
| **Bacteria (16S; V3-V4)** | | | | | | |
| GZ vs LNB | 1 | 0.466 | 0.466 | 5.85 | 0.594 | 0.0833 |
| GZ vs N | 1 | 0.473 | 0.473 | 9.41 | 0.702 | 0.0833 |
| GZ vs NB | 1 | 0.369 | 0.369 | 6.23 | 0.609 | 0.167 |
| LNB vs N | 1 | 0.344 | 0.344 | 4.64 | 0.537 | 0.167 |
| LNB vs NB | 1 | 0.329 | 0.329 | 3.97 | 0.498 | 0.0833 |
| N vs NB | 1 | 0.14 | 0.14 | 2.62 | 0.396 | 0.167 |
| All_groups | 3 | 1.06 | 0.353 | 5.31 | 0.666 | 0.0433 |
| **Fungi (ITS1)** | | | | | | |
| GZ vs LBN | 1 | 0.546 | 0.546 | 10.9 | 0.731 | 0.100 |
| GZ vs N | 1 | 0.501 | 0.501 | 8.5 | 0.68 | 0.100 |
| GZ vs NB | 1 | 0.385 | 0.385 | 9.57 | 0.705 | 0.100 |
| LBN vs N | 1 | 0.374 | 0.374 | 5.44 | 0.576 | 0.100 |
| LBN vs NB | 1 | 0.41 | 0.41 | 8.19 | 0.672 | 0.100 |
| N vs NB | 1 | 0.164 | 0.164 | 2.78 | 0.41 | 0.100 |
| All_groups | 3 | 1.19 | 0.397 | 7.28 | 0.732 | 0.001 |
| **Here,** soil fumigation with lime **(NB)**, soil fumigation with ammonium bicarbonate **(N)**, combined soil fumigation with ammonium bicarbonate and lime **(LNB)**, and non-fumigated soil **(GZ)**. | | | | | | |

| **Table S5:** Alpha diversity indices of 16S rRNA and ITS gene for bacterial and fungal communities under different treatments at the 97% similarity level. | | | | | |
| --- | --- | --- | --- | --- | --- |
| **Sample ID** | **Chao1** | **Simpson** | **Shannon** | **PD_whole_tree** | **Goods_coverage** |
| **Bacteria (16S; V3-V4)** | | | | | |
| NB | 3878.03±275.21ab | 0.0097±0.00377a | 2.6767±0.14844a | 118.04±9.08ab | 0.9886±0.00057a |
| N | 3944.30±43.31ab | 0.0085±0.00033a | 2.6767±0.01528a | 120.51±0.56ab | 0.9898±0.00033a |
| LNB | 4431.23±592.66a | 0.0472±0.07346a | 2.6400±0.53731a | 142.73±21.32a | 0.9887±0.00124a |
| GZ | 3328.73±44.12b | 0.0426±0.03679a | 2.3233±0.14189a | 100.93±1.39b | 0.9896±0.00042a |
| **Fungi (ITS1)** | | | | | |
| NB | 441.93±21.07a | 0.2123±0.03863a | 1.2667±0.09074a | 36.71±2.19b | 0.9963±0.0054a |
| N | 469.50±33.55a | 0.2179±0.18387a | 1.2787±0.35599a | 39.06±1.60ab | 0.9968±0.0074a |
| LNB | 485.60±24.65a | 0.0878±0.02209a | 1.5900±0.06083a | 40.88±2.12a | 0.9955±0.0048a |
| GZ | 473.80±20.63a | 0.0962±0.02412a | 1.4833±0.09609a | 38.26±1.47ab | 0.9968±0.0066a |
| **Here,** soil fumigation with lime **(NB)**, soil fumigation with ammonium bicarbonate **(N)**, combined soil fumigation with ammonium bicarbonate and lime **(LNB)**, and non-fumigated soil **(GZ)**. | | | | | |

| **Table S6:** Relative abundance of the top 15 dominant bacterial phyla in rhizosphere soil under different experimental conditions. (±SEM, n=3) | | | | | |
| --- | --- | --- | --- | --- | --- |
| Phylum | NB | N | LNB | GZ | Average (%) |
| Proteobacteria | 48.30±4.29c | 53.37±6.80b | 52.01±17.41b | 57.63±6.14a | 52.83 |
| Bacteroidetes | 25.46±8.23a | 25.63±6.22a | 16.62±5.16c | 20.81±6.67b | 22.13 |
| Acidobacteria | 7.63±1.80b | 4.58±0.63c | 10.18±4.86a | 7.58±0.31b | 7.49 |
| Actinobacteria | 3.92±0.61a | 4.13±0.61a | 3.45±1.02ab | 2.71±0.20b | 3.55 |
| Firmicutes | 4.36±1.35a | 2.21±0.43b | 3.38±0.94ab | 4.41±0.70a | 3.59 |
| Gemmatimonadetes | 2.86±0.83a | 1.73±0.31a | 3.02±0.86a | 1.90±0.36a | 2.38 |
| Chloroflexi | 2.22±0.72b | 1.94±0.18b | 3.55±1.82a | 1.25±0.17c | 2.24 |
| Patescibacteria | 1.53±0.57b | 2.66±0.33a | 1.42±0.47b | 0.72±0.16b | 1.58 |
| Verrucomicrobia | 1.22±0.29a | 1.33±0.53a | 2.19±1.42a | 0.97±0.17a | 1.41 |
| (Unassigned) | 0.95±0.10ab | 0.81±0.11ab | 1.26±0.41a | 0.74±0.50b | 0.94 |
| Nitrospirae | 0.60±0.13ab | 0.35±0.07b | 0.74±0.28a | 0.57±0.04ab | 0.57 |
| Armatimonadetes | 0.21±0.05ab | 0.17±0.02b | 0.34±0.11a | 0.25±0.05ab | 0.24 |
| Dependentiae | 0.11±0.02b | 0.40±0.11a | 0.45±0.13a | 0.04±0.01b | 0.25 |
| Planctomycetes | 0.17±0.08b | 0.17±0.01b | 0.41±0.21a | 0.11±0.02b | 0.22 |
| Chlamydiae | 0.09±0.03ab | 0.16±0.03a | 0.15±0.07a | 0.01±0.01b | 0.10 |
| others | 0.35±0.10b | 0.36±0.03b | 0.81±0.36a | 0.28±0.02b | 0.45 |
| **Here,** soil fumigation with lime **(NB)**, soil fumigation with ammonium bicarbonate **(N)**, combined soil fumigation with ammonium bicarbonate and lime **(LNB)**, and non-fumigated soil **(GZ)**. | | | | | |

| **Table S7:** Relative abundance of the top 15 dominant fungal phyla in rhizosphere soil under different experimental conditions. (±SEM, n=3) | | | | | |
| --- | --- | --- | --- | --- | --- |
| Phylum | NB | N | LNB | GZ | Average (%) |
| Olpidiomycota | 44.33±4.51a | 41.07±21.26a | 23.76±4.96b | 21.76±5.58b | 32.73 |
| Ascomycota | 15.51±4.51c | 22.88±5.79b | 13.54±2.65c | 27.49±7.16a | 19.86 |
| Nematoda | 5.78±1.71b | 9.02±4.83ab | 14.04±4.20a | 4.32±0.17b | 8.29 |
| Ciliophora | 6.15±2.29a | 6.07±4.36a | 8.51±4.34a | 11.12±5.43a | 7.96 |
| Arthropoda | 4.45±6.20a | 2.11±2.25a | 3.70±5.04a | 5.24±1.45a | 3.88 |
| (Unassigned) | 4.27±0.86b | 4.51±1.74b | 16.08±1.01a | 4.64±0.17b | 7.38 |
| Mortierellomycota | 7.24±0.25a | 2.23±0.89c | 2.75±0.63c | 5.23±1.55b | 4.36 |
| Plasmodiophoromycota | 0.69±0.18b | 0.06±0.03b | 0.08±0.04b | 16.30±2.13a | 4.28 |
| Basidiomycota | 4.58±2.07a | 3.89±1.65ab | 2.90±0.47b | 1.24±0.40c | 3.15 |
| Chytridiomycota | 3.42±1.24ab | 4.07±2.73a | 2.10±0.43b | 0.97±0.43c | 2.64 |
| Ochrophyta | 0.23±0.06b | 0.53±0.28a | 0.39±0.04ab | 0.25±0.06ab | 0.35 |
| unidentified | 1.56±0.44a | 1.78±0.79a | 1.60±0.54a | 0.80±0.45a | 1.44 |
| GS01 | 0.00±0.00b | 0.00±0.00b | 6.63±2.04a | 0.00±0.00b | 1.66 |
| Rozellomycota | 0.99±0.76a | 0.93±0.21a | 1.68±0.25a | 0.06±0.04b | 0.92 |
| Mucoromycota | 0.48±0.08a | 0.31±0.15a | 1.32±1.73a | 0.29±0.12a | 0.60 |
| others | 0.31±0.09b | 0.50±0.46ab | 0.91±0.09a | 0.27±0.15b | 0.50 |
| **Here,** soil fumigation with lime **(NB)**, soil fumigation with ammonium bicarbonate **(N)**, combined soil fumigation with ammonium bicarbonate and lime **(LNB)**, and non-fumigated soil **(GZ)**. | | | | | |

| **Table S8:** Relative abundance of the top 15 dominant bacterial genera in rhizosphere soil under different experimental conditions. (±SEM, n=3) | | | | | |
| --- | --- | --- | --- | --- | --- |
| Genus | NB | N | LNB | GZ | Average (%) |
| *(Unassigned)* | 21.36±3.65a | 20.89±0.80a | 27.01±9.35a | 32.74±12.12a | 25.50 |
| *Flavobacterium* | 13.66±9.12a | 11.52±5.35b | 3.64±0.84a | 4.11±3.26a | 8.23 |
| *uncultured* | 7.56±1.95a | 7.24±0.71a | 9.11±3.29a | 5.32±0.70a | 7.31 |
| *Pseudomonas* | 4.82±2.59b | 4.71±1.06b | 3.80±2.13b | 7.93±4.04a | 5.32 |
| *Sphingomonas* | 4.96±0.52ab | 4.62±0.64ab | 5.63±2.20a | 3.54±0.71b | 4.69 |
| *Allorhizobium* | 3.22±0.38b | 4.37±0.76a | 1.60±0.39c | 3.20±0.25b | 3.10 |
| *Bacillus* | 3.95±1.25a | 1.85±0.39b | 2.90±0.89ab | 3.43±0.65ab | 3.03 |
| *Providencia* | 0.03±0.01a | 0.03±0.01a | 11.87±20.52a | 0.08±0.04a | 3.00 |
| *Pedobacter* | 2.78±0.94ab | 3.51±0.42a | 0.32±0.08c | 2.01±0.78b | 2.16 |
| *Sphingobacterium* | 0.15±0.03b | 0.36±0.12b | 0.05±0.02b | 3.79±1.75a | 1.09 |
| *Flavisolibacter* | 1.81±0.33a | 1.01±0.11b | 1.86±0.69a | 2.05±0.31a | 1.68 |
| *Stenotrophomonas* | 0.26±0.05b | 0.83±0.27b | 0.07±0.01b | 3.86±1.47a | 1.26 |
| *Bryobacter* | 1.16±0.19ab | 0.90±0.08b | 1.58±0.55a | 1.27±0.05ab | 1.23 |
| *Luteimonas* | 1.70±0.66ab | 2.60±0.91a | 0.80±0.27bc | 0.14±0.05c | 1.31 |
| *Acidovorax* | 1.07±0.32ab | 1.25±0.11a | 0.40±0.10b | 1.22±0.67a | 0.99 |
| *others* | 31.51±5.06ab | 34.30±3.01a | 29.37±5.13ab | 25.31±2.91b | 30.12 |
| **Here,** soil fumigation with lime **(NB)**, soil fumigation with ammonium bicarbonate **(N)**, combined soil fumigation with ammonium bicarbonate and lime **(LNB)**, and non-fumigated soil **(GZ)**. | | | | | |

| **Table S9:** Relative abundance of the top 15 dominant fungal genera in rhizosphere soil under different experimental conditions. (±SEM, n=3) | | | | | |
| --- | --- | --- | --- | --- | --- |
| Genus | NB | N | LNB | GZ | Average (%) |
| *(Unassigned)* | 33.99±1.84b | 44.14±15.75ab | 58.62±3.60a | 36.25±4.04b | 43.25 |
| *Olpidium* | 44.34±4.51a | 41.08±21.27a | 23.74±4.93b | 21.79±5.62b | 32.74 |
| *unidentified* | 5.25±1.12b | 4.10±1.56b | 6.03±1.05b | 10.14±1.78a | 6.38 |
| *Plasmodiophora* | 0.69±0.18b | 0.06±0.03c | 0.08±0.04c | 16.32±2.12a | 4.29 |
| *Mortierella* | 6.27±0.33a | 2.02±0.78b | 2.11±0.82b | 2.83±0.75b | 3.31 |
| *Fusarium* | 3.68±1.57a | 2.14±0.44b | 1.07±0.43c | 2.96±0.47ab | 2.46 |
| *Acrobeloides* | 0.63±0.52a | 2.72±2.22a | 0.49±0.74a | 0.61±0.22a | 1.11 |
| *Tetrahymena* | 0.00±0.00b | 0.02±0.01b | 0.27±0.40ab | 3.08±3.00a | 0.84 |
| *Latorua* | 0.14±0.04c | 0.59±0.27b | 1.23±0.16a | 0.02±0.02c | 0.50 |
| *Solicoccozyma* | 0.67±0.65a | 0.15±0.13a | 0.32±0.11a | 0.80±0.15a | 0.49 |
| *Cylindrocarpon* | 0.03±0.02b | 0.01±0.01b | 0.04±0.03b | 1.68±1.11a | 0.44 |
| *Actinomucor* | 0.22±0.06a | 0.06±0.05a | 0.87±1.40a | 0.18±0.14a | 0.33 |
| *Powellomyces* | 0.17±0.12a | 0.08±0.02ab | 0.03±0.03b | 0.01±0.01b | 0.07 |
| *Ceratophysella* | 0.02±0.01a | 0.07±0.10a | 0.01±0.01a | 0.00±0.00a | 0.03 |
| *Spumella* | 0.03±0.03a | 0.01±0.01a | 0.03±0.02a | 0.01±0.01a | 0.02 |
| *others* | 3.86±1.66a | 2.72±1.17a | 5.05±1.02a | 3.32±1.41a | 3.74 |
| **Here,** soil fumigation with lime **(NB)**, soil fumigation with ammonium bicarbonate **(N)**, combined soil fumigation with ammonium bicarbonate and lime **(LNB)**, and non-fumigated soil **(GZ)**. | | | | | |

| **Table S10:** Relative abundance of the top 20 dominant bacterial OTUs in rhizosphere soil under different experimental conditions. (±SEM, n=3) | | | | | |
| --- | --- | --- | --- | --- | --- |
| OTUs | NB | N | LNB | GZ | Average (%) |
| OTU_3 | 3.02±1.15a | 1.38±0.35b | 2.15±0.64ab | 2.57±0.42ab | 2.28 |
| OTU_27 | 2.65±3.04a | 0.72±0.28a | 0.10±0.03a | 1.48±2.18a | 1.24 |
| OTU_12 | 1.06±0.31ab | 1.22±0.11a | 0.37±0.07b | 1.21±0.67a | 0.97 |
| OTU_14 | 0.81±0.57b | 1.32±0.75b | 0.75±0.10b | 4.76±2.82a | 1.91 |
| OTU_5 | 0.64±0.27b | 0.15±0.03b | 0.28±0.10b | 15.55±11.23a | 4.16 |
| OTU_4 | 0.14±0.02b | 0.19±0.11b | 0.05±0.00b | 3.35±1.15a | 0.93 |
| OTU_9 | 0.09±0.02b | 0.20±0.07b | 0.02±0.01b | 3.29±1.61a | 0.90 |
| OTU_1 | 0.03±0.01a | 0.03±0.01a | 11.84±20.47a | 0.08±0.04a | 2.99 |
| OTU_73 | 0.64±0.17b | 0.72±0.13b | 1.53±0.53a | 0.51±0.03b | 0.85 |
| OTU_2 | 3.27±0.62a | 2.98±0.46a | 2.91±1.19a | 2.30±0.48a | 2.86 |
| OTU_11 | 3.27±1.74a | 2.79±0.57a | 2.80±2.07a | 1.89±0.63a | 2.69 |
| OTU_33 | 1.79±1.79a | 1.76±1.42a | 0.04±0.01a | 0.70±0.91a | 1.07 |
| OTU_7 | 2.05±0.66b | 2.87±0.25a | 0.18±0.06c | 0.78±0.26c | 1.47 |
| OTU_37 | 0.72±0.20b | 1.19±0.34a | 0.33±0.11b | 0.49±0.09b | 0.68 |
| OTU_17 | 0.98±0.77b | 1.99±0.61a | 0.69±0.25b | 0.27±0.09b | 0.98 |
| OTU_6 | 1.90±0.25b | 2.59±0.42a | 1.22±0.29c | 1.16±0.22c | 1.72 |
| OTU_40 | 1.57±0.58ab | 2.45±0.85a | 0.66±0.23bc | 0.09±0.02c | 1.19 |
| OTU_18 | 1.07±0.23a | 1.42±0.49a | 0.51±0.19b | 0.38±0.09b | 0.85 |
| OTU_400 | 0.66±0.25a | 1.12±0.43a | 0.76±0.24a | 0.77±0.19a | 0.83 |
| OTU_32 | 0.59±0.16bc | 1.35±0.45a | 0.83±0.24b | 0.22±0.04c | 0.75 |
| **Here,** soil fumigation with lime **(NB)**, soil fumigation with ammonium bicarbonate **(N)**, combined soil fumigation with ammonium bicarbonate and lime **(LNB)**, and non-fumigated soil **(GZ)**. | | | | | |

| **Table S11:** Relative abundance of the top 20 dominant fungal OTUs in rhizosphere soil under different experimental conditions. (±SEM, n=3) | | | | | |
| --- | --- | --- | --- | --- | --- |
| OTUs | NB | N | LNB | GZ | Average (%) |
| OTU_14 | 3.92±2.14a | 1.10±0.23b | 0.83±0.35b | 4.09±1.73a | 2.48 |
| OTU_15 | 1.17±0.97ab | 0.44±0.28b | 0.76±0.58ab | 1.83±0.49a | 1.05 |
| OTU_18 | 0.92±0.33b | 0.56±0.17bc | 0.42±0.07c | 1.57±0.24a | 0.87 |
| OTU_37 | 0.01±0.01b | 0.01±0.01b | 0.37±0.44b | 3.92±1.04a | 1.08 |
| OTU_23 | 0.78±0.45b | 0.63±0.18b | 0.58±0.09b | 1.62±0.43a | 0.90 |
| OTU_3 | 0.69±0.18b | 0.06±0.03b | 0.08±0.04b | 16.32±2.12a | 4.29 |
| OTU_5 | 0.84±0.10b | 0.26±0.03b | 0.23±0.16b | 8.97±6.22a | 2.58 |
| OTU_9 | 4.21±2.31b | 4.98±2.98b | 11.11±3.29a | 2.99±0.22b | 5.82 |
| OTU_17 | 0.05±0.04b | 0.67±0.24b | 4.79±3.62a | 0.36±0.09b | 1.47 |
| OTU_24 | 0.91±0.36b | 0.89±0.44b | 5.36±0.68a | 0.80±0.17b | 1.99 |
| OTU_13 | 0.00±0.00b | 0.00±0.00b | 5.61±1.88a | 0.00±0.01b | 1.40 |
| OTU_25 | 1.18±0.36a | 1.34±0.58a | 0.97±0.46ab | 0.22±0.08b | 0.93 |
| OTU_2 | 44.34±4.51a | 41.08±21.27a | 23.74±4.93a | 21.79±5.62a | 32.74 |
| OTU_16 | 1.67±0.69a | 1.57±0.80a | 0.68±0.10ab | 0.02±0.02b | 0.98 |
| OTU_20 | 1.15±0.13ab | 1.43±0.52a | 0.21±0.18c | 0.76±0.31bc | 0.89 |
| OTU_4 | 1.89±0.37b | 7.04±2.76a | 1.13±0.40b | 0.50±0.32b | 2.64 |
| OTU_7 | 0.88±0.22b | 7.16±2.21a | 0.36±0.19b | 0.07±0.02b | 2.11 |
| OTU_21 | 3.81±6.57a | 0.00±0.00a | 2.95±5.10a | 0.00±0.00a | 1.69 |
| OTU_6 | 3.43±1.50a | 2.06±0.43ab | 0.96±0.40b | 2.22±0.10ab | 2.17 |
| OTU_51 | 4.40±0.54a | 0.26±0.23b | 0.53±0.14b | 0.36±0.05b | 1.39 |
| **Here,** soil fumigation with lime **(NB)**, soil fumigation with ammonium bicarbonate **(N)**, combined soil fumigation with ammonium bicarbonate and lime **(LNB)**, and non-fumigated soil **(GZ)**. | | | | | |
